# HIV, malnutrition, and noncommunicable disease epidemics among tuberculosis-affected households in east and southern Africa: A cross-sectional analysis of the ERASE-TB cohort

**DOI:** 10.1371/journal.pmed.1004452

**Published:** 2024-09-16

**Authors:** Claire Jacqueline Calderwood, Edson Tawanda Marambire, Leyla Larsson, Denise Banze, Alfred Mfinanga, Celina Nhamuave, Tejawsi Appalarowthu, Mishelle Mugava, Jorge Ribeiro, Peter Edwin Towo, Karlos Madziva, Justin Dixon, Kathrin Held, Lilian Tina Minja, Junior Mutsvangwa, Celso Khosa, Norbert Heinrich, Katherine Fielding, Katharina Kranzer, ERASE-TB consortium

**Affiliations:** 1 Clinical Research Department, London School of Hygiene & Tropical Medicine, London, United Kingdom; 2 The Health Research Unit Zimbabwe, Biomedical Research and Training Institute, Harare, Zimbabwe; 3 CIH^LMU^ Center for International Health, University Hospital, LMU Munich, Munich, Germany; 4 Division of Infectious Diseases and Tropical Medicine, University Hospital, LMU Munich, Munich, Germany; 5 Instituto Nacional de Saúde (INS), Marracuene, Mozambique; 6 National Institute for Medical Research–Mbeya Medical Research Centre, Mbeya, Tanzania; 7 Department of Global Health and Development, London School of Hygiene & Tropical Medicine, London, United Kingdom; 8 German Centre for Infection Research (DZIF), partner site Munich, Germany; 9 Fraunhofer Institute for Translational Medicine and Pharmacology ITMP, Immunology, Infection and Pandemic Research, Munich, Germany; 10 Biomedical Research and Training Institute, Harare, Zimbabwe; 11 TB Centre, London School of Hygiene & Tropical Medicine, London, United Kingdom

## Abstract

**Background:**

As a result of shared social and structural risk factors, people in households affected by tuberculosis may have an increased risk of chronic conditions; at the same time, tuberculosis screening may be an opportunity for interventions. We sought to describe the prevalence of HIV, nutritional disorders, and noncommunicable diseases (NCDs) among members of tuberculosis-affected households in 3 African countries.

**Methods and findings:**

A part of a multicountry cohort study, we screened for tuberculosis, HIV, nutritional disorders (underweight, anaemia, overweight/obesity), and NCDs (diabetes, hypertension, and chronic lung disease) among members of tuberculosis-affected households aged ≥10 years in Mozambique, Tanzania, and Zimbabwe. We describe the prevalence of these conditions, their co-occurence within individuals (multimorbidity) and household-level clustering. Of 2,109 household contacts recruited, 93% (*n* = 1,958, from 786 households) had complete data and were included in the analysis. Sixty-two percent were female, median age was 27 years, and 0.7% (*n* = 14) were diagnosed with co-prevalent tuberculosis. Six percent of household members (*n* = 120) had previous tuberculosis, 15% (*n* = 294) were living with HIV, 10% (*n* = 194) had chronic lung disease, and 18% (*n* = 347) were anaemic. Nine percent of adults (*n* = 127) had diabetes by HbA1c criteria, 32% (*n* = 439) had hypertension. By body mass index criteria, 18% household members (*n* = 341) were underweight while 29% (*n* = 549) were overweight or obese. Almost half the household members (*n* = 658) had at least 1 modifiable tuberculosis risk factor. Sixty-one percent of adults (*n* = 822) had at least 1 chronic condition, 1 in 4 had multimorbidity. While most people with HIV knew their status and were on treatment, people with NCDs were usually undiagnosed and untreated. Limitations of this study include use of point-of-care HbA1c for definition of diabetes and definition of hypertension based on single-day measurements.

**Conclusions:**

Households affected by tuberculosis also face multiple other health challenges. Integrated approaches to tuberculosis screening may represent an opportunity for identification and treatment, including prioritisation of individuals at highest risk for tuberculosis to receive preventive therapy.

## Abstract 2

Claire J. Calderwood and colleagues examine the prevalence of HIV, malnutrition, and non-communicable diseases among households with a member affected by tuberculosis in East and Southern Africa.

## Author summary

**Why was this study done?:**

- Tuberculosis-affected households are a particular priority group for interventions to address infectious, nutritional, and noncommunicable causes of illness and death. These household members may be at particular risk of these conditions due to underlying structural determinants of health, while the opportunity to intervene presented by tuberculosis screening may enable chronic conditions to be addressed, reducing tuberculosis incidence and improving overall health.
- Few studies have reported on the prevalence of conditions other than HIV among members of TB-affected households, therefore the burden of infectious, nutritional, and noncommunicable diseases within TB-affected households is unknown.

**What did the researchers do and find?:**

- As part of a cohort study of people living in tuberculosis-affected households in 3 east/southern African countries, we screened 1,958 adolescents and adults for tuberculosis, HIV, nutritional disorders (including underweight, anaemia, overweight/obesity), and noncommunicable diseases (NCDs; diabetes, hypertension, and chronic lung disease).
- We found a high prevalence of these conditions and identified a very large gap in access to care for NCDs. While some conditions clustered within households (HIV, undernutrition), others (NCDs) did not.
- One in 2 tuberculosis household contacts had a modifiable tuberculosis risk factor (HIV, diabetes, chronic lung disease, underweight, current smoker, or potential alcohol misuse) while one in 5 had 2 or more chronic conditions (i.e., multimorbidity), most commonly due to the coexistence of HIV and an NCD.

**What do these findings mean?:**

- In Africa, households affected by tuberculosis are impacted not only by tuberculosis but by multiple colliding epidemics including HIV, malnutrition, and NCDs.
- Screening for tuberculosis among household contacts represents an opportunity to intervene, improving the health of a vulnerable community while preventing tuberculosis. There is now clear evidence, including that from this study, to justify such an approach.
- Implementation research including health economic evaluations are now needed to support guideline development and scale up.

## Introduction

The epidemiological transition model describes a shift in population-level burden of disease from infections and undernutrition to noncommunicable diseases (NCDs) due to social, economic, and demographic change. However, the reality is often a “double” or “triple” burden of disease; resulting from an increased prevalence of NCDs, and associated risk factors, and a slow decline or persistence of infectious diseases and malnutrition [1]. This collision of epidemics is reinforced by shared underlying structural and social determinants of health including poverty, low health literacy, and lack of universal health coverage [2]. Consequently, the impact is greatest among socioeconomically vulnerable communities who can least afford the consequences of ill health [3,4].

Chronic conditions, including HIV, diabetes, undernutrition, and behavioural risk factors such as alcohol use disorder and smoking are the major attributable causes of tuberculosis globally [5]. While transmission of diseases such as tuberculosis and HIV commonly occurs within households, households also share behavioural (e.g., diet, smoking, physical activity) and genetic characteristics. As a result, members of tuberculosis-affected households may be at increased risk of both infectious and noncommunicable chronic conditions; however, the existing evidence is limited. In Africa, previous studies have described the prevalence of HIV (systematic revew of 13 studies; pooled prevalence 7.3% [6]) or diabetes (*n* = 3 studies, prevalence range 5.2% to 17.4% [7–9]). No previous studies from Africa have explicitly evaluated prevalence of undernutrition, obesity, or chronic lung disease among members of tuberculosis-affected households and none have considered more than one of these conditions or described their overlap in individuals and households.

Systematic screening for tuberculosis among household contacts may present an opportunity to identify and treat chronic conditions and risk factor clusters, reducing progression to tuberculosis and improving overall health [9–11].

As part of a multinational, observational, prospective cohort study (Early Risk Assessment in tuberculosis contactS by new diagnostic tEsts [ERASE-TB]), we sought to describe the prevalence of HIV, nutritional disorders, and NCDs among members of tuberculosis-affected households in 3 African countries. We hypothesised that the prevalence of these conditions would be high, that most NCDs would be undiagnosed or untreated, and that there would be evidence for clustering of chronic conditions at household level.

## Methods

### ERASE-TB study overview

The ERASE-TB study protocol has been published [12]. In brief, the study consists of 3 primary and 3 secondary objectives. The primary ERASE-TB objectives relate to determination of diagnostic accuracy of novel tuberculosis tests to diagnose TB earlier, using prospective cohort data. The secondary objectives are (1) to determine *Mycobacterium tuberculosis* infection prevalence among household contacts at baseline and during follow-up; (2) to establish a biorepository of cryopreserved specimens for future development and validation of diagnostic tests; and (3) to assess the prevalence of selected chronic diseases among household contacts and their association with tuberculosis. Secondary objective 3 is addressed in the present manuscript.

The study population is people aged at least 10 years old and living with someone with recently diagnosed, highly infectious, microbiologically confirmed (Xpert MTB/RIF “medium” or higher) pulmonary tuberculosis, enrolled at 3 sites (Maputo, Mozambique; Mbeya, Tanzania; and Harare, Zimbabwe; recruitment: March 2021 to March 2023) and followed up every 6 months for up to 24 months (projected end date: December 2024). We use “household contacts” to differentiate these individuals from the person with tuberculosis whose diagnosis resulted in recruitment of the household. This analysis followed a prespecified analysis plan (February 2022; S1 Appendix) and uses data collected at baseline or within the first 12 months of follow up, if missing at baseline (S1 Appendix). Thus data contributing to this analysis were collected between March 2021 and March 2024. The sample size was determined by the primary objective of determining sensitivity and specificity of novel tuberculosis diagnostics.

### Definition of chronic conditions

Baseline data were used for analysis. If NCD testing was not done at baseline, it was performed at a subsequent timepoint (Table A in S1 Appendix). Tuberculosis screening comprised a World Health Organization (WHO) symptom screen and chest X-ray, followed by Xpert MTB/Rif Ultra (Cepheid, USA) if either suggested tuberculosis. Point-of-care HIV testing followed national guidelines and height and weight were measured for calculation of body mass index (BMI). Handheld spirometry (EasyOne Air or Easy on-PC) was performed according to American Thoracic Society/European Respiratory Society (ATS/ERS) standards [13]. Among adults aged 18 years and older, glycated haemoglobin (HbA1c) (A1c Care, SD Biosensor) testing was offered and blood pressure assessed according to the WHO STEPwise approach to surveillance protocol [13]. An interviewer-administered questionnaire included demographics, medical history, smoking history, and the alcohol use disorders identification test short-form (AUDIT-C) [14]. Conditions were categorised using internationally agreed definitions (Tables B–D in S1 Appendix). While we use the terms diabetes and hypertension, these are based on screening on a single day; we acknowledge that this does not fulfil diagnostic criteria [15,16]. Chronic lung disease was defined as previously diagnosed lung disease or an obstructive or mixed defect on pre-bronchodilator spirometry, interpreted using ATS/ERS standards with Global Lung Initiative “other” reference (S1 Appendix). Absolute BMI (adults) and BMI-for-age Z-scores (adolescents) were used to create combined categories; notably BMI-for-age Z-score <-2 among adolescents (termed “thinness” per WHO) was categorised as moderate/severe underweight (Table C in S1 Appendix). Multimorbidity was defined as 2 or more chronic conditions in one individual [17]. Participants screening positive for any condition were referred according to local standards of care.

### Statistical analysis

Statistical analyses were performed in R (version 4.2.2); packages are detailed in S1 Appendix. Analyses are reported in accordance with Strengthening the Reporting of Observational studies in Epidemiology (STROBE) guidelines (S1 STROBE Checklist). Following description of missing data (Table F in S1 Appendix), a complete case records analysis was conducted. All analyses, aside from analyses characterising household-level clustering (below) were adjusted for household-level clustering using design-based standard errors.

The number of household contacts with symptoms or chest X-ray suggestive of tuberculosis, and the number diagnosed with co-prevalent tuberculosis was described. Subsequent analyses focused on other chronic conditions (HIV, diabetes, hypertension, anaemia, chronic lung disease), BMI category, and stunting. For each of these, overall prevalence and prevalence stratified by age category (adolescent [10, 17 years], younger adult [18,39], and older adult [40 and older]), sex and study site were calculated. Prevalence and associated 95% confidence intervals (95% CI) were additionally stratified by sex and age-standardised to the WHO reference population using direct standardization to aid comparisons. HIV prevalence was additionally calculated stratified by HIV status of the person with tuberculosis as an exploratory analysis. Following peer review, we additionally considered prevalence of chronic conditions, stratified by area of residence (rural, per-urban, and urban) within the Tanzanian site where participants across these areas were included.

Among adults, a cascade of care was constructed for HIV, diabetes, and hypertension. This described the proportion of people with the condition who knew their status, the proportion of those with known status who were on treatment, and the proportion of people of those who had “disease control.” Disease control was defined as CD4 >350 cells/μl for HIV, HbA1c <6.5% for diabetes, and systolic BP <140 and diastolic BP <90 for hypertension. HIV viral loads were not available.

We explored the coexistence of conditions within individuals through intersection plots, and the trajectory of nutritional indices across years of age through calculation of age-specific Z-scores among both adolescents and adults (S1 Appendix).

The clustering of conditions within households was described by constructing a multilevel model including each condition, in turn, as the outcome, no individual-level explanatory variables, and a random-effect for household. The variation partition coefficient (VPC) and *p*-value for a random-effect at household level was considered as a measure of the residual variation that is attributable to unobserved household characteristics, with the VPC being equivalent to the intra-cluster correlation coefficient.

### Sensitivity analyses

Recognising the insufficiency of single-day blood pressure measurements to confirm hypertension [16,18] and in view of the very high prevalence of hypertension (based on a single-day reading) in our dataset, we calculated the proportion of people categorised as “screening detected” hypertension who were confirmed through a second elevated blood pressure measurement at the subsequent follow-up visit.

### Ethical considerations

Informed written consent was obtained from all participants aged 18 years and older; individual assent and guardian consent was obtained from participants aged 10 to 17 years. Ethical approval was granted by all relevant institutions: the Medical Research Council in Zimbabwe (MRCZ/A/2618), the Mbeya Medical Research and Ethics Review Committee (SZEC-2439/R.A/V.1/101), the National Health Research Ethics Committee in Tanzania (NIMR/HQ/R.8a/Vol.IX/3608), and Tanzanian Medicines and Medical Devices Authority (TMDA-WEB0021/CTR/0004/03), the National Bioethics Committee for Health in Mozambique (541/CNBS/21), and the ethical committees of London School of Hygiene & Tropical Medicine, United Kingdom (22522–2) and the medical faculty of the Ludwig Maximilian-Universität München, Germany (20–0771).

## Results

### Participant characteristics

**Fig 1:**
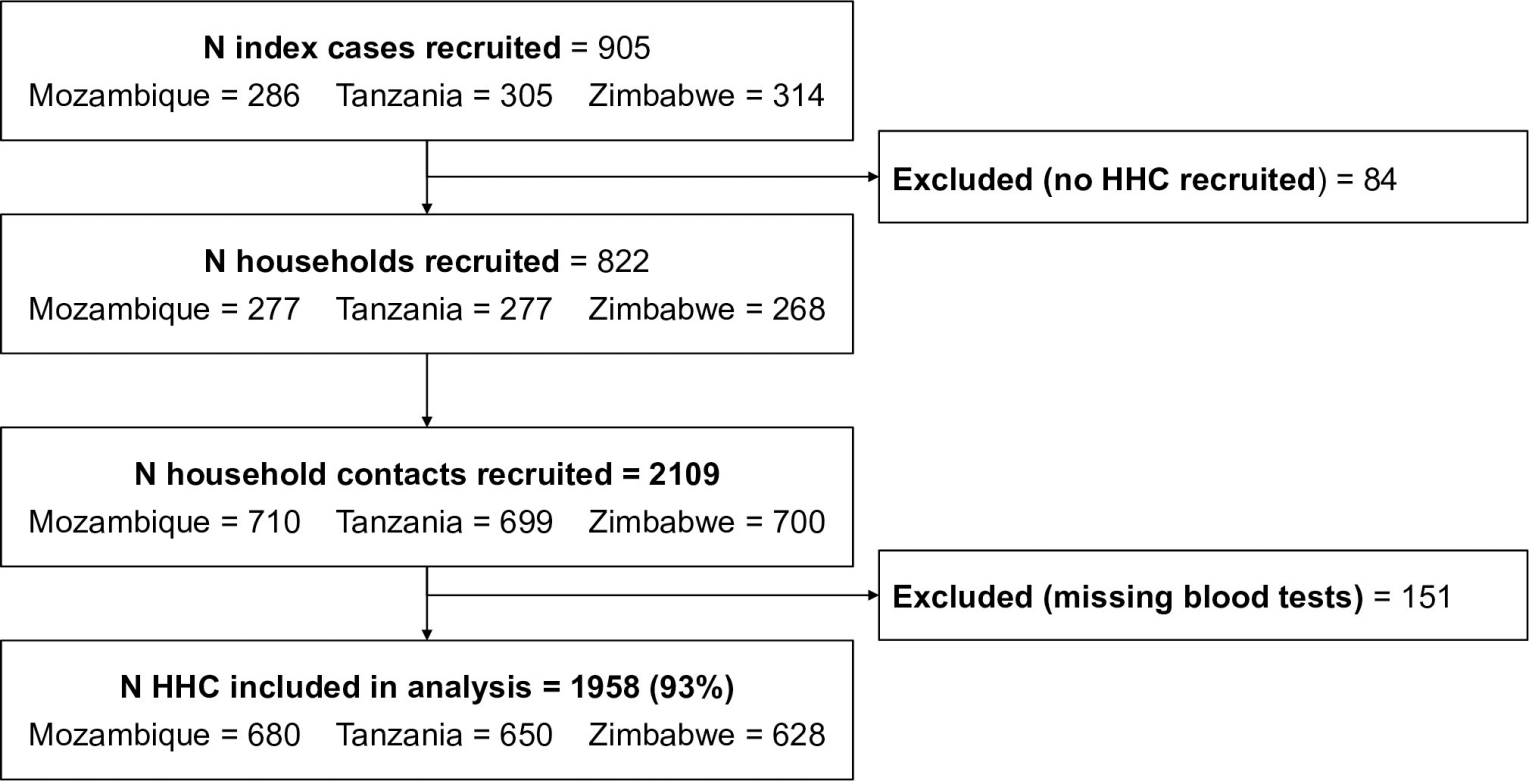
Flow diagram illustrating recruitment of participants and inclusion in analysis data set. HHC, household contacts; N, number.

Between March 2021 and March 2023, 2,649 household contacts were identified from 905 people with TB (median 3 [interquartile range (IQR) 2, 5]; 2 [IQR 2, 3] and 2 [IQR 1, 4] per household in Mozambique, Tanzania, and Zimbabwe, respectively). Of these, 2,109 (80%) from 822 households were recruited, with a median of 2 contacts recruited per household in each country (Mozambique, IQR 1, 4; Tanzania and Zimbabwe, IQR 1, 3). The maximum household size recruited was 18 people. No participants were missing an HIV result, 2 participants were missing BMI, fewer than 1.8% of people were missing results for each of CD4 and BP; 3.6% people were missing HbA1c and 2.7% people were missing haemoglobin (Table F in S1 Appendix). A total of 1,958 people (93%; 680 (96%) in Mozambique, 650 (93%) in Tanzania, and 628 (90%) in Zimbabwe) from 786 households were included in the analysis (Fig 1). Demographic details for included and excluded participants are shown in Table G in S1 Appendix.

Among included participants, 62% (*n* = 1,217) were female and the median age was 27 years (IQR 16, 42), with 31% of household contacts (*n* = 602) being adolescents aged 10 to 17 years (Table 1). Men were, on average, younger than women (median 21 [IQR 14, 37] years versus 30 [18, 45] years): 22% of women (*n* = 267) were spouses of people with tuberculosis, compared to 8.5% men (*n* = 63), resulting in more adult women than men (Table H and Fig A in S1 Appendix). Smoking was relatively infrequent, with 7.1% participants (16% of men and 1.4% women) ever having smoked, the median pack-year smoking exposure was 1.8 (IQR 0.6, 4.6) years. Overall, 33% people (5.3% [*n* = 32] adolescents) reported drinking alcohol; 14% people (*n* = 265; 21% of men [*n* = 155], 9.0% of women [*n* = 11] and 1.5% of adolescents [*n* = 9]) screened positive for hazardous use of alcohol using AUDIT-C.

**Table 1:** Characteristics of study population (*N* = 1,958).

Characteristic	Overall *N* = 1,958	Mozambique *N* = 680	Tanzania *N* = 650	Zimbabwe *N* = 628
**Sex**				
Women	1,217 (62%)	411 (60%)	416 (64%)	390 (62%)
Men	741 (38%)	269 (40%)	234 (36%)	238 (38%)
**Age, years**	27 (16, 42)	23 (16, 39)	29 (16, 44)	28 (17, 42)
**Age category**				
10–17 years	602 (31%)	221 (33%)	203 (31%)	178 (28%)
18–39 years	807 (41%)	298 (44%)	235 (36%)	274 (44%)
40+ years	549 (28%)	161 (24%)	212 (33%)	176 (28%)
**Highest educational level** *				
None or primary school	897 (48%)	253 (39%)	472 (75%)	172 (29%)
At least secondary school	973 (52%)	390 (61%)	156 (25%)	427 (71%)
**Pregnant** †	38 (3.1%)	8 (1.9%)	14 (3.4%)	16 (4.1%)
**Smoking status**				
Non smoker	1,819 (93%)	654 (96%)	612 (94%)	553 (88%)
Smoker (current and/or former)	138 (7.1%)	26 (3.8%)	38 (5.8%)	74 (12%)
**Pack years smoking**	1.8 (0.6, 4.6)	1.8 (1.2, 3.6)	1.5 (0.5, 4.2)	2.1 (0.8, 5.8)
**Alcohol consumption**				
Never drunk alcohol	1,308 (67%)	437 (64%)	425 (65%)	446 (71%)
Alcohol, AUDIT-C negative	384 (20%)	162 (24%)	124 (19%)	98 (16%)
Alcohol, AUDIT-C positive	265 (14%)	80 (12%)	101 (16%)	84 (13%)
**Insufficient food** ‡	535 (27%)	241 (35%)	38 (5.8%)	256 (41%)
**Relationship to index case**				
Spouse	330 (17%)	109 (16%)	123 (19%)	98 (16%)
Parent	499 (25%)	193 (28%)	165 (25%)	141 (22%)
Sibling	368 (19%)	134 (20%)	118 (18%)	116 (18%)
Child	267 (14%)	90 (13%)	91 (14%)	86 (14%)
Other	494 (25%)	154 (23%)	153 (24%)	187 (30%)

Presented as number (percentage) or median (interquartile range).

*Educational level not known for 88 participants.

^†^The denominator for pregnancy is the number of women.

^‡^Insufficient food was defined as participants as answering yes to “was there any day in the past 6 months where you did not have enough food.”

AUDIT-C, alcohol use identification test, short form; TB, tuberculosis.

Socioeconomic deprivation was common: 20% of households were crowded (*n* = 157; defined as at least 3 people per room) and 90% (*n* = 583) were under the international poverty line (income less than United States Dollar 1.90 per person per day; Table 2). In 41% (*n* = 321) of households, the primary income earner was the person who had tuberculosis. Food insecurity varied by site: in Mozambique and Zimbabwe (majority urban/peri-urban) 35% and 41% people, respectively, reported food insecurity, compared to 5.8% in Tanzania (where the proportion of peri-urban and rural participants was higher).

**Table 2:** Characteristics of study households (*N* = 786*)*.

Characteristic	Overall *N* = 786	Mozambique *N* = 269	Tanzania *N* = 264	Zimbabwe *N* = 253
***N* people in household** *	5 (3, 6)	6 (4, 7)	4 (3, 6)	5 (3, 6)
**Crowding** *	157 (20%)	64 (24%)	18 (6.8%)	75 (30%)
**Household income per person per day, USD** †	0.64 (0.34, 1.09)	0.66 (0.41, 1.03)	0.42 (0.23, 0.85)	0.74 (0.41, 1.56)
**Primary earner is the person with TB**	321 (41%)	68 (26%)	137 (52%)	116 (46%)
**Household income <1.90 USD per person per day**	583 (90%)	209 (90%)	171 (95%)	203 (86%)
**Area of residence**				
Rural	103 (13%)	5 (1.9%)	97 (37%)	1 (0.4%)
Peri-urban	262 (33%)	196 (73%)	56 (21%)	10 (4.0%)
Urban	420 (54%)	68 (25%)	110 (42%)	242 (96%)
**HIV status of person with TB** ‡				
Negative	499 (68%)	173 (68%)	172 (71%)	154 (65%)
Positive, on ART	154 (21%)	57 (22%)	46 (19%)	51 (21%)
Positive, not on ART	82 (11%)	24 (9.4%)	25 (10%)	33 (14%)

Presented as number (percentage) or median (interquartile range).

*The total number (*N*) of people in a household includes both people eligible for the study and children under the age of 10, who were not eligible. Crowding using UN definition (≥3 people per room).

^†^Income not known for 137 households. A threshold of 1.90 USD was used as this was the international poverty line threshold in 2021 when data collection began. In September 2022, the threshold was increased to 2.15 USD.

^‡^HIV status not known for 51 people with TB.

ART, anti-retroviral therapy; *N*, number; TB, tuberculosis; USD, United States Dollars.

### Chronic conditions among tuberculosis household contacts

Overall, 6% (*n* = 120) people had a previous history of tuberculosis. Three hundred and seventy-five people (20%) were screened as possible tuberculosis (322 had symptoms suggestive of tuberculosis and 87 had a CXR suggestive of tuberculosis on review by a clinical officer; 34 had both criteria met). Fourteen people were diagnosed with co-prevalent tuberculosis: 13 had a positive Xpert and 1 was diagnosed through chest X-ray.

Prevalence of HIV was 15.0% (95% confidence interval [95% CI] 13.2, 17.0%; *n* = 294), with 13 people (4.4%; 95% CI 2.6, 7.4%) with HIV having advanced disease (CD4 count less than 200 cells/μl; Table 3; confidence intervals are shown in Table I in S1 Appendix). Table J and Fig C in S1 Appendix shows prevalences by site. Prevalence of HIV was higher among women compared to men (19.1% [16.7, 21.6%; *n* = 232] versus 8.4% [6.4, 10.8%; *n* = 62]), although this difference was partially attenuated in age-standardised prevalence estimates (19.7% [17.3%, 22.0%] versus 12.5% [8.7%, 16.4%]; Table 4). While HIV prevalence was low among adolescents (*n* = 16, 2.7% [1.6, 4.3%]), 7/16 adolescents with HIV were not aware of their status. When stratified by the HIV status of the index case, 9.8% (95% CI 8.0, 12.0%; *n* = 121) household contacts of HIV–negative index cases had HIV (0.9% [*n* = 11] were diagnosed through screening) compared to 25.2% (95% CI 21.2, 29.6%; *n* = 143) household contacts of HIV–positive index cases (2.8% [*n* = 16] were diagnosed through screening; Table L in S1 Appendix).

**Table 3:** Chronic conditions among tuberculosis household contacts (*N* = 1,958).

Condition	Level	Overall	Women	Men	10–17 years	18–39 years	40+ years
All participants		*N* = 1,958	*N* = 1,217	*N* = 741	*N* = 602	*N* = 807	*N* = 549
HIV prevalence		15.0%	19.1%	8.4%	2.7%	14.3%	29.7%
CD4 category*	≥500 cells/μl	63.5%	66.7%	51.6%	76.5%	59.6%	64.8%
	200–499 cells/μl	32.1%	29.9%	40.3%	23.5%	36.8%	29.7%
	<200 cells/μl	4.4%	3.4%	8.1%		3.5%	5.5%
Median CD4 (cells/μl)*		584	603	526	678	565	589
BMI category†	Moderate/severe underweight	3.9%	2.5%	6.1%	8.1%	2.2%	1.6%
	Mild underweight	13.5%	9.6%	20.0%	27.4%	9.2%	4.7%
	Healthy weight	54.5%	49.8%	62.3%	57.0%	59.4%	44.8%
	Overweight	17.4%	22.0%	9.7%	6.3%	18.8%	27.3%
	Obese	10.7%	16.0%	1.9%	1.2%	10.4%	21.5%
Chronic lung disease prevalence‡		10.3%	11.0%	9.2%	9.1%	9.4%	13.0%
Anaemia prevalence§		17.7%	20.7%	12.8%	16.6%	18.0%	18.6%
Anaemia category§	None	82.3%	79.3%	87.2%	83.4%	82.0%	81.4%
	Mild anaemia	11.5%	12.2%	10.3%	11.8%	10.7%	12.4%
	Moderate anaemia	5.6%	7.6%	2.4%	4.3%	6.6%	5.6%
	Severe anaemia	0.6%	0.9%	0.1%	0.5%	0.7%	0.5%
Hb (g/dL)		135	130	144		137	135
**Adolescents**		***N* = 602**	***N* = 305**	***N* = 297**	***N* = 602**		
Stunting^ll^	Normal	56.6%	62.3%	50.8%	56.6%		
	Mild stunting	26.7%	26.2%	27.3%	26.7%		
	Moderate stunting	12.8%	8.9%	16.8%	12.8%		
	Severe stunting	3.8%	2.6%	5.1%	3.8%		
**Adults**		***N* = 1,356**	***N* = 912**	***N* = 444**		***N* = 807**	***N* = 549**
Diabetes prevalence		9.4%	10.4%	7.2%		4.8%	16.0%
HbA1c category	<6.0%	62.8%	62.5%	63.3%		68.9%	53.7%
	6.0–6.4%	28.8%	27.9%	30.9%		27.1%	31.3%
	6.5–6.9%	5.3%	6.0%	3.8%		2.7%	9.1%
	≥7.0%	3.1%	3.6%	2.0%		1.2%	5.8%
HbA1c (%)		5.8	5.8	5.8		5.7	5.9
Hypertension prevalence		32.4%	35.3%	26.4%		16.1%	56.3%
BP category	Normal BP	54.8%	53.7%	57.0%		71.9%	29.7%
	High-normal BP	15.6%	14.6%	17.6%		14.1%	17.7%
	Grade 1 HTN	16.3%	16.9%	15.1%		9.0%	27.0%
	Grade 2 HTN	13.3%	14.8%	10.4%		5.0%	25.7%
Systolic BP (mmHg)		119.5	120	119		120	137
Diastolic BP (mmHg)		76.5	78	74		77	86.5

Values are presented as percentages (for categorical variables) or medians (for continuous variables). “Prevalent” disease is defined as either known or screening detected.

*CD4 counts are only reported among people living with HIV, excluding 5 people with missing CD4 count (*N* = 293).

^†^BMI categories were created using BMI for age Z-scores among adolescents (<19 years) with WHO references and absolute BMI thresholds among adults (≥19 years). Mod/severe underweight = Z-score <-2 or BMI < 17 kg/m^2^; mild underweight = Z-score ≥-2 and <-1 or BMI 17–18.49 kg/m^2^; normal weight = Z-score ≥-1 and ≤+1 or BMI 18.5–24.9 kg/m^2^; overweight = Z-score >+1 and ≤+2 or BMI 25–29.9 kg/m^2^; obese = Z-score >+2 or BMI >30.0 kg/m^2^.

^‡^Chronic lung disease was defined as either a previous diagnosis, or an obstructive or mixed defect on pre-bronchodilator spirometry (less than lower limit of normal using Global Lung Initiative “Other” reference standard). 80.8% (*n* = 1,582) participants had an adequate quality spirometry, those missing spirometry traces were categorised as not having a chronic lung disease (unless self-reported).

^§^Anaemia was categorised using age and sex-specific thresholds recommended by WHO (S1 Appendix).

^ll^Stunting was only calculated among adolescents, using WHO reference standards. Mild stunting was defined as a height of age Z-score of <-1 and ≥-2; moderate stunting as Z-score of <-2 and ≥-3 and severe stunting as Z-score <-3.

BMI, body mass index; BP, blood pressure; Hb, haemoglobin; HbA1c, glycosylated haemoglobin; HTN, hypertension; *N*, number; WHO, World Health Organization.

**Table 4:** Prevalence of chronic conditions among tuberculosis household contacts, standardised to the World Health Organization reference population.

Condition*	Overall	Women	Men
HIV	16.9% (14.7%, 19.1%)	19.7% (17.3%, 22.0%)	12.5% (8.7%, 16.4%)
Diabetes	9.6% (8.0%, 11.3%)	10.8% (8.7%, 13.0%)	7.6% (5.1%, 10.0%)
Hypertension	36.8% (34.2%, 39.3%)	39.1% (36.2%, 41.9%)	33.0% (28.6%, 37.4%)
Anaemia	16.7% (15.0%, 18.5%)	20.0% (17.7%, 22.3%)	11.5% (9.0%, 14.0%)
Underweight	15.2% (13.4%, 16.9%)	9.6% (8.3%, 11.0%)	23.9% (20.0%, 27.8%)
Overweight/Obese	30.5% (28.5%, 32.6%)	40.1% (37.4%, 42.8%)	15.4% (12.5%, 18.3%)
Chronic lung disease	10.4% (8.6%, 12.2%)	10.2% (8.4%, 12.0%)	10.8% (7.4%, 14.2%)

*N = 1958 overall, 1217 women and 741 men; other than for diabetes and hypertension where prevalence was calculated among adults only (N = 1356, 912 women and 444 men). 95% confidence intervals (presented in brackets) were calculated using design-based standard errors within the ‘survey’ package in R.

A total of 127 adults had diabetes (9.4% [7.9, 11.1%]) and 439 had hypertension (32.4% [29.8, 35.1%]). A further 28.8% of adults (*n* = 391) had an HbA1c of between 6.0% and 6.5% while 15.6% (*n* = 211) had a blood pressure in the high-normal range. The prevalence of HIV, diabetes, and hypertension increased with age, with a linear association between each of HbA1c, systolic and diastolic blood pressure and age (Figs D and E in S1 Appendix). The prevalence of chronic lung disease was 10.3% (8.9, 11.9%; *n* = 194), similar across sex and slightly higher in older adults.

Overall, 17.7% people were at least mildly underweight (95% CI 15.6, 19.4%; *n* = 341); 3.9% were moderately or severely underweight (3.0, 4.9%; *n* = 76); 17.4% people were overweight (95% CI 15.7, 19.2%; *n* = 340) and a further 10.7% were obese (95% CI 9.4, 12.2%; *n* = 209). More adolescents and men were underweight, while overweight and obesity were more common among older adults and women (Fig F in S1 Appendix). BMI-for-age Z-scores increased with age, notably among women between the ages of 10 and 40 years. Prevalence of anaemia was 17.7% (95% CI 15.9, 19.7%; *n* = 347), higher among women than men.

**Fig 2:**
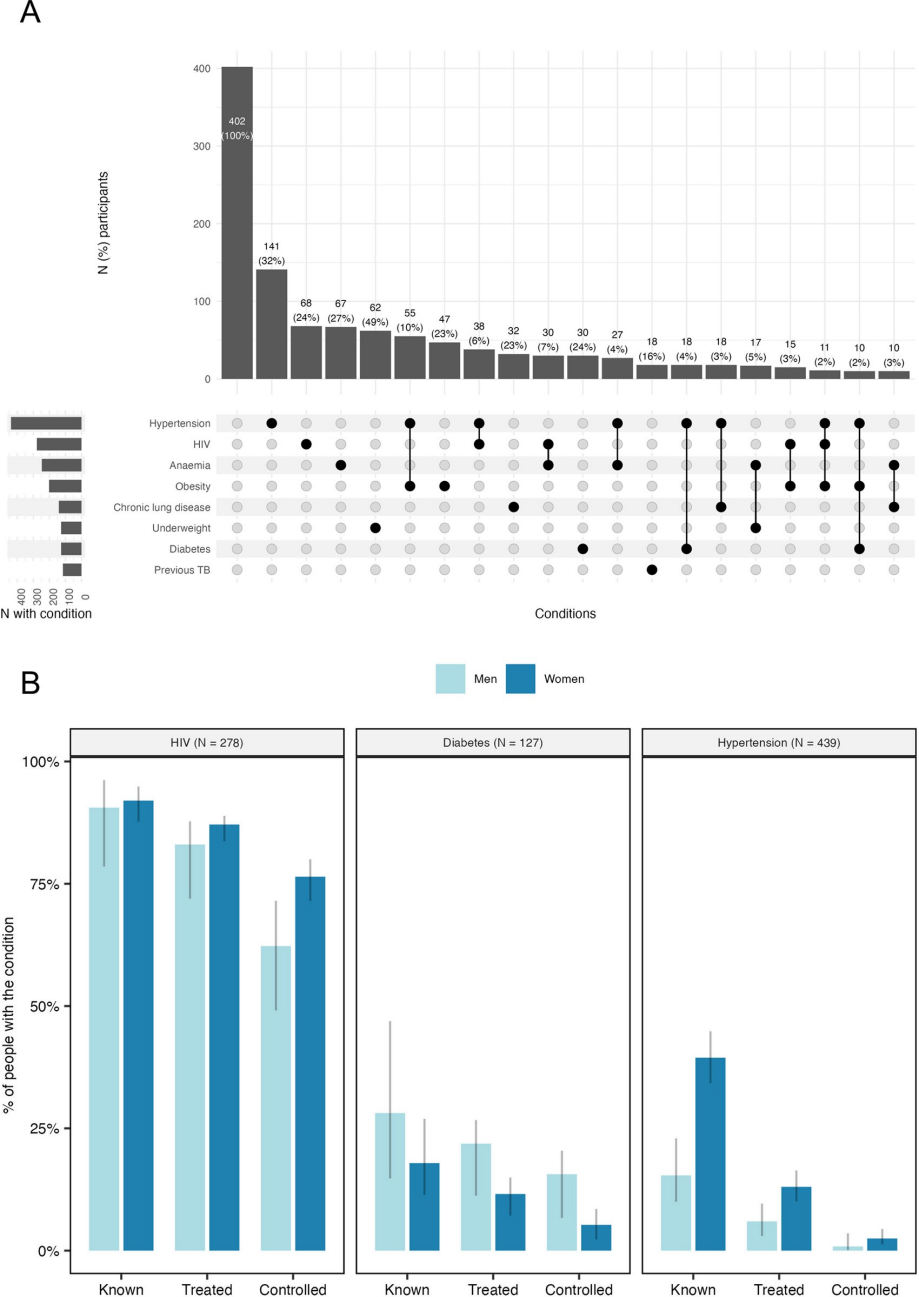
Intersection of chronic conditions (A) and cascade of care for HIV, diabetes, and hypertension (B) among adult tuberculosis household contacts (*N* = 1,356). For panel A, Intersections are exclusive (i.e., individuals are only represented in the graph once) and only intersections containing at least 10 individuals are shown. The bottom sub-panel shows the included chronic conditions (y axis), with the left section indicating the overall number (*N*) participants with each and the right section showing the specific disease combinations (represented by black circles). The top sub-panel shows intersection size, i.e., the percentage and number of participants with each disease combination. For panel B, known (disease) was defined as a self-report of having, or being on medication for a condition; treated was defined as being on a relevant medication for the condition; controlled was defined as, for HIV, CD4 count >350 cells/μl, for diabetes, an HbA1c of <6.5% and for hypertension, as a blood pressure of <140 mmHg systolic and <90 mmHg diastolic. These are presented as relative proportions of the total number of people with each condition (100%, with number of people with disease [*N*] shown in sub-panel header), stratified by sex, with their 95% confidence intervals (grey lines).

Forty-nine percent of adults (*n* = 658) and 38% adolescents (*n* = 228) had at least 1 modifiable tuberculosis risk factor (HIV, diabetes, underweight, current smoker, or AUDIT score suggestive of alcohol use). Sixty-one percent of adults (*n* = 822) had at least 1 chronic condition (any of HIV, diabetes, hypertension, chronic lung disease, or anaemia); 24% (*n* = 326) had multimorbidity (2 or more of the above). The most common multimorbidity was of hypertension and HIV (10%; Fig 2A). In Mbeya (Tanzania), prevalence of NCDs appeared higher in peri-urban and rural areas, compared to urban residents, albeit with overlapping confidence intervals (Tables O and P in S1 Appendix).

Overall, 81% adults (*n* = 1,179) had blood pressure readings from at least 1 subsequent study visit and were included in the sensitivity analysis of “confirmed” hypertension, with 58.8% percent of people (*n* = 151/257) with hypertension detected through screening at baseline having this confirmed. If 2 consecutive elevated blood were required for criteria for hypertension to be met, the prevalence of hypertension among adult household contacts was 19.6% (*n* = 227).

### Cascade of care for chronic conditions

Fig 2B and Table M in S1 Appendix illustrate the cascade of care for adults with HIV, diabetes, and hypertension. The majority of people with HIV knew their status (91.7% of those with HIV; 95% CI 87.7, 94.5%; *n* = 255), were on treatment (95.2% of those with known status; 95% CI 91.8, 97.3%; *n* = 240), and had a CD4 count of at least 350 cells/μl (85.4% of those on treatment; 95% CI 80.1, 89.5%; *n* = 205). Among people with diabetes, 20.5% were aware of this (95% CI 14.5, 28.2%; *n* = 26), 69.2% of those were on treatment (95% CI 49.1, 84.0%; *n* = 18), and 55.6% of those had controlled disease (32.7, 76.3%; *n* = 10). Among people with hypertension the corresponding figures were 33.0% (28.8, 37.6%; *n* = 145), 33.8% (26.7, 41.6%; *n* = 49), and 18.4% (9.8, 31.8%; *n* = 9). The gap in diagnosis for hypertension and diabetes appeared larger in Tanzania than the other 2 sites (Fig G in S1 Appendix).

### Clustering within households

In keeping with the individual-level analysis, household-level prevalence of chronic conditions was high. Almost a third of households (31%, *n* = 245) had at least 1 member with HIV, half (48%, *n* = 379) had 1 member with an NCD (Table N in S1 Appendix), and a third (32%, *n* = 253) had at least 1 member who was underweight. Eleven percent of households included both someone who was underweight and someone who was overweight or obese (Fig H in S1 Appendix).

There was strong evidence for correlation within households for HIV, anaemia, and underweight status (Table 5). The highest variation particion coefficient (VPC) was for HIV (42%) suggesting that almost half the variation in HIV prevalence was due to household characteristics; the lowest VPC was for hypertension (9.2%) suggesting that the majority of variation in hypertension prevalence was due to individual-level rather than household-level variation.

**Table 5:** Household-level clustering of chronic conditions.

	Fixed effect model*	Mixed effects model*			
	Prevalence (95% CI)	Prevalence (95% CI)^†^	Variance	VPC^‡^	p value^§^
**All participants (N = 1958)**					
HIV^ll^	15.0% (13.2, 17.0%)	9.4% (7.2, 12.1%)	2.38	42.0%	<0.001
Anaemia	17.7% (15.9, 19.7%)	13.7% (11.5, 16.4%)	1.13	25.5%	<0.001
Underweight	17.4% (15.6, 19.4%)	14.0% (11.8%, 16.6%)	0.79	19.3%	<0.001
**Adults (N = 1356)**					
Diabetes	9.4% (7.9, 11.1%)	7.4% (5.0, 10.6%)	0.69	17.4%	0.06
Hypertension	32.4% (29.8, 35.1%)	30.9% (27.9, 34.1%)	0.33	9.2%	0.02

* The fixed effect model is a single-level model including only the outcome. The mixed effects model additionally included a random effect for household. In the fixed effect model, 95% confidence intervals (95% CI) are adjusted for household level using robust standard errors.

† Prevalence in the mixed effects model is calculated from the intercept and equivalent to a geometric mean.

‡ The variance partition coefficient (VPC) is an estimate of how much residual variation in prevalence is due to unobserved level 2 (household) characteristics.

§ p value for a random effect at level 2 (household level).

^ll^Restricting HIV analysis to adults only (to aid comparison with diabetes and hypertension) Fixed effect model: prevalence 20.5% (18.3, 23.1%), Mixed effects model: prevalence 12.9% (9.7, 17.0%), variance 3.07, VPC 48.2%, p < 0.001.

## Discussion

This study demonstrated a high prevalence of HIV, diabetes, hypertension, anaemia, and chronic lung disease among household contacts of people with tuberculosis in Mozambique,Tanzania, and Zimbabwe. While awareness of and effective treatment for HIV was high, most people with diabetes and hypertension were unaware of their status, those who were diagnosed were often un- or suboptimally treated. Over a third of adolescents were at least mildly underweight. By contrast, fewer adults were underweight, while overweight and obesity was very common, particularly among women. Together, these data demonstrate that households are affected not only by tuberculosis but by multiple colliding epidemics which increase the risk of tuberculosis and poor tuberculosis-specific outcomes, and result in other poor health outcomes.

There are very limited data on the prevalence of chronic conditions among tuberculosis household contacts, with this being the first study to simultaneously consider more than one infectious and noninfectious chronic condition. Previous studies from Africa have described the prevalence of single diseases including HIV (pooled prevalence from 13 studies 7.3% [3.6, 11.1%] [6]) and diabetes (2 studies: Ethiopia [prevalence 5.2%] and South Africa [prevalence 17.4%; 44% undiagnosed] [7,8]) among tuberculosis household contacts. One study compared tuberculosis household contacts to community controls, finding an elevated risk of HIV in the former [19]. For other chronic conditions, whether there is a higher risk of chronic conditions among members of tuberculosis-affected households compared to others living in the same community is unknown. Here, to provide some insights into this, we calculated age-standardised prevalence to facilitate comparisons with published estimates (Tables E and K in S1 Appendix). While prevalence of most conditions was similar, underweight appeared more common among our population compared to country-specific national-level estimates. We also found strong evidence of correlation within households in HIV status, underweight and anaemia but not NCDs. In other words, household members appear to resemble each other in terms of HIV, anaemia, and underweight, but less so in terms of hypertension or diabetes. This may suggest that, while tuberculosis-affected households have a specific risk of HIV (due to transmission within households) and undernutrition (due to shared diet), drivers of NCDs do not act at household level. The high prevalence of NCDs in our study population, under this interpretation, reflects tuberculosis-burden communities being at high risk of other health conditions due to shared structural and social determinants of health, rather than a specific increased risk among these households compared to matched community controls [20]. Assuming that household members are genetically similar, it also suggests the most important drivers of NCDs are environmental and structural, not genetic. Tuberculosis can therefore be considered an “indicator” of a high-risk community, at the centre of biosocial syndemics, rather than reflecting a direct biological association between tuberculosis and risk of NCDs. While comparison of tuberculosis-affected household members to community controls may provide further insights, the high burden of disease demonstrated here, together with the opportunity presented by tuberculosis screening to intervene are, in our view, sufficient to justify inclusion of interventions addressing upstream determinants of tuberculosis during household contact screening.

Strengths of our study include the large, multi-centre design, with objective screening for a range of conditions, rather than relying on self-report which can severely underestimate disease prevalence. This also enabled us to quantify the gap in access to care. Most eligible household contacts identified participated, limiting selection bias. While most of our participants were women, the gender-balance of households when including the person with tuberculosis was even, suggesting this reflects the majority of people with tuberculosis being men (in keeping with local tuberculosis epidemiology) rather than underparticipation by men. This was facilitated by flexible visits, including weekends and holidays, supporting participation by people in work.

Limitations of this study include a relatively limited assessment of nutritional status and use of point-of-care HbA1c, which may underestimate diabetes prevalence [21]. We used measurement of blood pressure on a single day as the primary definition of hypertension to align with the WHO STEPs protocol and other screening studies; our sensitivity analysis suggested this may have overestimated the absolute prevalence of confirmed hypertension by 12.8%. This is similar to results elsewhere [18]. Our definition of chronic lung disease was limited to obstructive airways or self-reported disease and therefore chronic lung diseases not included in this definition may have been missed. While participation by eligible household contacts was high overall, we cannot exclude that household contacts with health concerns were more likely to participate, resulting in overestimation of prevalence of chronic diseases. Data were missing for some assessments due to delayed implementation of chronic disease screening in the study owing to delays in shipping of consumables and ethical approvals in the context of COVID-19. There was no evidence that the people with missing data were systematically different to those who were included; this is unlikely to have resulted in bias.

Recently, the potential impact of addressing upstream determinants to prevent tuberculosis and improve outcomes was demonstrated in a large cluster-randomised trial in India, where provision of macro- and micro-nutrient support to tuberculosis household contacts reduced tuberculosis incidence by 40% [10]. In that trial, undernutrition was very common (e.g., 40% among adult women), while few people were overweight or obese. In our context, where both undernutrition and overweight/obesity coexist, it is likely that a locally developed intervention including a nuanced communication strategy will be needed [22]. This double burden of malnutrition is likely to become increasingly relevant for tuberculosis programmes as economic globalisation reaches the poorest communities worldwide, resulting in a shift in the burden of overweight/obesity from the richest to the poorest, while undernutrition persists [23].

The very high risk of tuberculosis among people with HIV is well recognised [5]. In our study yield of screening among contacts of people with tuberculosis and HIV was higher than among people without HIV. People with diabetes have up to 3 times elevated risk of tuberculosis compared to those without [24], while achieving diabetic control may reduce tuberculosis risk [25]. Screening for diabetes among tuberculosis household contacts, together with prompt treatment, could improve outcomes in people with co-prevalent tuberculosis and prevent incident tuberculosis [26,27]. In the context of slow rollout of tuberculosis preventive therapy, and low tuberculosis preventive therapy completion, identification of people with conditions that increase the risk of tuberculosis may also facilitate prioritisation of the highest risk household contacts for tuberculosis preventive therapy initiation and adherence support. However, the resource implications of chronic disease screening within tuberculosis screening interventions need to be evaluated.

The gap in access to care described in this study is not specific to tuberculosis-affected households: as east and southern African countries approach or exceed the 95-95-95 targets for HIV, it is estimated that two-thirds of people with diabetes and half of people with hypertension do not know their status, while half of those with a diagnosis are not on effective treatment [28,29]. While recognising the need for population-wide interventions to prevent, diagnose, and treat NCDs, leveraging strong, disease-specific programmes to provide a wider range of services may be one approach to expanded service provision [11]. This is gaining traction with differentiated service delivery for other chronic diseases within HIV care, and recommendations for screening for diabetes, HIV, and undernutrition among people with tuberculosis [30,31]. In the context of preexisting financial insecurity, the catastrophic healthcare expenditure associated with tuberculosis is likely to exacerbate already extremely high barriers to accessing healthcare, particularly care for NCDs which, in Africa, are usually associated with high clinic, medication, and other costs [32]. Households affected by tuberculosis divert resources to support the person who is unwell; this is likely to include delaying health seeking and interrupting treatment for chronic conditions where that will cut costs. In order to improve outcomes, it is imperative that screening interventions among high-tuberculosis burden communities include clear pathways to onward care, and support for the financial costs of accessing care and adhering to treatment.

Overall, our study shows, for the first time, that tuberculosis household contacts in east and southern Africa are living with a range of chronic health concerns, many of which are associated with an increased risk of tuberculosis. Recognising that barriers to healthcare are often greatest for poor and vulnerable communities, the most recent WHO guidance on systematic screening for tuberculosis recommends that “where possible, community screening should be combined with screening for other diseases or risk factors and with health-promotion or social support activities” [33]. Aligned to this goal, our study supports integration of screening for other conditions among tuberculosis household contacts in Africa. Such an approach may reduce tuberculosis incidence and reduce the consequences of poor health among a community who can least afford them.

## Supporting information

S1 STROBE Checklist Completed STROBE checklist. (PDF)

S1 Acknowledgements Membership of the ERASE-TB consortium. (PDF)

S1 Protocol Study protocol. (PDF)

S1 Appendix Supplementary methods and results. (PDF)

## Abbreviations

95%CI: 95% confidence interval
BMI: body mass index
HbA1c: glycated haemoglobin
IQR: interquartile range
NCD: noncommunicable disease
VPC: variation partition coefficient
WHO: World Health Organization
