# Supplementary material for: HIV, malnutrition, and noncommunicable disease epidemics among tuberculosis-affected households in east and southern Africa: A cross-sectional analysis of the ERASE-TB cohort
Source: PLoS Med. 2024 Sep 16;21(9):e1004452. doi: 10.1371/journal.pmed.1004452 (PMC11441706; doi:10.1371/journal.pmed.1004452)
Supplement: S1 Acknowledgements — (PDF) [file pmed.1004452.s002.pdf]

ERASE-TB consortium

Norbert	Heinrich	Division of Infectious Diseases and Tropical Medicine, University Hospital, LMU Munich, Munich, Germany<br>German Center for Infection Research, Partner Site Munich, Munich, Germany<br>Fraunhofer Institute for Translational Medicine and Pharmacology ITMP, Immunology, Infection and Pandemic Research, Munich, Germany
Ursula	Panzer	Division of Infectious Diseases and Tropical Medicine, University Hospital, LMU Munich, Munich, Germany<br>German Center for Infection Research, Partner Site Munich, Munich, Germany<br>Fraunhofer Institute for Translational Medicine and Pharmacology ITMP, Immunology, Infection and Pandemic Research, Munich, Germany
Christof	Geldmacher	Division of Infectious Diseases and Tropical Medicine, University Hospital, LMU Munich, Munich, Germany<br>German Center for Infection Research, Partner Site Munich, Munich, Germany<br>Fraunhofer Institute for Translational Medicine and Pharmacology ITMP, Immunology, Infection and Pandemic Research, Munich, Germany
Kathrin	Held	Division of Infectious Diseases and Tropical Medicine, University Hospital, LMU Munich, Munich, Germany<br>German Center for Infection Research, Partner Site Munich, Munich, Germany<br>Fraunhofer Institute for Translational Medicine and Pharmacology ITMP, Immunology, Infection and Pandemic Research, Munich, Germany
Andrea	Rachow	Division of Infectious Diseases and Tropical Medicine, University Hospital, LMU Munich, Munich, Germany<br>German Center for Infection Research, Partner Site Munich, Munich, Germany<br>Fraunhofer Institute for Translational Medicine and Pharmacology ITMP, Immunology, Infection and Pandemic Research, Munich, Germany
Friedrich	Rieß	Division of Infectious Diseases and Tropical Medicine, University Hospital, LMU Munich, Munich, Germany
Tejaswi	Appalarowthu	Division of Infectious Diseases and Tropical Medicine, University Hospital, LMU Munich, Munich, Germany
Deepika	Zende	Division of Infectious Diseases and Tropical Medicine, University Hospital, LMU Munich, Munich, Germany
Leyla	Larsson	Division of Infectious Diseases and Tropical Medicine, University Hospital, LMU Munich, Munich, Germany
Adrian	Ruhle	Division of Infectious Diseases and Tropical Medicine, University Hospital, LMU Munich, Munich, Germany
Lilian Tina	Minja	National Institute for Medical Research - Mbeya Medical Research Centre, Mbeya, Tanzania
Nyanda	Elias Ntinginya	National Institute for Medical Research - Mbeya Medical Research Centre, Mbeya, Tanzania
Issa	Sabi	National Institute for Medical Research - Mbeya Medical Research Centre, Mbeya, Tanzania
Alfred	Mfinanga	National Institute for Medical Research - Mbeya Medical Research Centre, Mbeya, Tanzania<br>CIH^{LMU} Center for International Health, University Hospital, LMU Munich, Munich, Germany
Harieth	Mwambola	National Institute for Medical Research - Mbeya Medical Research Centre, Mbeya, Tanzania
Elizabeth	Ntapara	National Institute for Medical Research - Mbeya Medical Research Centre, Mbeya, Tanzania
Harrieth	Mwambola	National Institute for Medical Research - Mbeya Medical Research Centre, Mbeya, Tanzania
Lwithio	Sudi	National Institute for Medical Research - Mbeya Medical Research Centre, Mbeya, Tanzania
Bariki	Mtafya	National Institute for Medical Research - Mbeya Medical Research Centre, Mbeya, Tanzania
Emanuel	Sichone	National Institute for Medical Research - Mbeya Medical Research Centre, Mbeya, Tanzania
Doreen	Pamba	National Institute for Medical Research - Mbeya Medical Research Centre, Mbeya, Tanzania
Abisai	Kisinda	National Institute for Medical Research - Mbeya Medical Research Centre, Mbeya, Tanzania
Peter	Towo	National Institute for Medical Research - Mbeya Medical Research Centre, Mbeya, Tanzania
Lilian	Njovu	National Institute for Medical Research - Mbeya Medical Research Centre, Mbeya, Tanzania
Alice	Shoo	National Institute for Medical Research - Mbeya Medical Research Centre, Mbeya, Tanzania
Willyhelmina	Olomi	National Institute for Medical Research - Mbeya Medical Research Centre, Mbeya, Tanzania
Celso	Khosa	Instituto Nacional de Saúde (INS), Marracuene, Mozambique
Denise Floripes	Banze	Instituto Nacional de Saúde (INS), Marracuene, Mozambique<br>CIH^{LMU} Center for International Health, University Hospital, LMU Munich, Munich, Germany
Nelson	Tembe	Instituto Nacional de Saúde (INS), Marracuene, Mozambique
Nadia	Sitoe	Instituto Nacional de Saúde (INS), Marracuene, Mozambique
Carla	Madeira	Instituto Nacional de Saúde (INS), Marracuene, Mozambique
Candido	Azize	Instituto Nacional de Saúde (INS), Marracuene, Mozambique
Celina	Nhamuave	Instituto Nacional de Saúde (INS), Marracuene, Mozambique

Sidonia	Nhacubangane	Instituto Nacional de Saúde (INS), Marracuene, Mozambique
Jorge	Ribeiro	Instituto Nacional de Saúde (INS), Marracuene, Mozambique
Junior	Mutsvangwa	Biomedical Research and Training Institute, Harare, Zimbabwe
Edson	Marambire	The Health Research Unit Zimbabwe, Biomedical Research and Training Institute, Harare, Zimbabwe<br>CIH[LMU] Center for International Health, University Hospital, LMU Munich, Munich, Germany
Fungai	Kavenga	Ministry of Health and Child Care, Harare, Zimbabwe
Tsitsi	Bandason	The Health Research Unit Zimbabwe, Biomedical Research and Training Institute, Harare, Zimbabwe
Kuda	Mutasa	Zvitambo Institute for Maternal and Child Health, Harare, Zimbabwe
Martha	Chipinduro	The Health Research Unit Zimbabwe, Biomedical Research and Training Institute, Harare, Zimbabwe
Sandra	Rukobo	Zvitambo Institute for Maternal and Child Health, Harare, Zimbabwe
Mishelle	Mugava	The Health Research Unit Zimbabwe, Biomedical Research and Training Institute, Harare, Zimbabwe
Beauty	Makamure	Biomedical Research and Training Institute, Harare, Zimbabwe
Forget	Makoga	Biomedical Research and Training Institute, Harare, Zimbabwe
Katharina	Kranzer	The Health Research Unit Zimbabwe, Biomedical Research and Training Institute, Harare, Zimbabwe<br>Division of Infectious Diseases and Tropical Medicine, Medical Center of the University of Munich, Munich, Germany<br>Clinical Research Department, London School of Hygiene & Tropical Medicine, London, UK<br>German Center for Infection Research (DZIF), Partner Site Munich, Munich, Germany
Claire	Calderwood	The Health Research Unit Zimbabwe, Biomedical Research and Training Institute, Harare, Zimbabwe<br>Clinical Research Department, London School of Hygiene & Tropical Medicine, London, UK
Hazel	Dockrell	London School of Hygiene & Tropical Medicine, London, UK
Anna	Shepherd	London School of Hygiene & Tropical Medicine, London, UK
Gunilla	Källenius	Department of Medicine, Karolinska Institutet, Stockholm, Sweden
Christopher	Sundling	Department of Medicine, Karolinska Institutet, Stockholm, Sweden
Lindsay	Zurba	Education for Health Africa, South Africa
