# Supplementary material for: HIV, malnutrition, and noncommunicable disease epidemics among tuberculosis-affected households in east and southern Africa: A cross-sectional analysis of the ERASE-TB cohort
Source: PLoS Med. 2024 Sep 16;21(9):e1004452. doi: 10.1371/journal.pmed.1004452 (PMC11441706; doi:10.1371/journal.pmed.1004452)
Supplement: S1 Protocol — (PDF) [file pmed.1004452.s003.pdf]

# Integrated multi-component health checks for TB-affected households (IMBA Hutano)

An evaluation of disease clustering and the acceptability and yield of integrated health screening in southern Africa

**Extract of study protocol (dated 1st February 2022)**

# Chronic diseases analysis

## Study design

This will comprise a primary data analysis, exploring the burden and patterns of multi-morbidity in ERASE-TB. It contributes to secondary objective 3 of the ERASE-TB study:

- To estimate the proportion of HHCs with chronic diseases (abnormal lung function, diabetes, hypertension and under-/overweight) at baseline and explore the relationship between chronic diseases and co-prevalent/incident TB.

<u>Ethical approvals</u>

Ethical approval for ERASE-TB has been granted by the ethics committees of LSHTM (22522) and University of Munich (20-0771) as well as national ethics committees in Mozambique (541/CNBS/21), Tanzania (NIMR/HQ/R.8a/Vol.IX/3608 and SZEC-2439/R.A/V.1/101) and Zimbabwe (MRCZ/A/2618). All above committees except Tanzania (review pending) have approved an amended protocol (V4.0) which covers the work outlined below.

## Study population

### ERASE-TB

This will be a comprehensive analysis of data collected for ERASE-TB (three sites; n=2100) which includes HIV testing, spirometry, digital chest radiograph (dCXR), height, weight and mid-upper arm circumference (MUAC). In addition, blood pressure, HbA1c and haemoglobin will be measured at the baseline study visit, or at follow up if not possible/missed at baseline, for the purposes of this analysis. Previous diagnoses of chronic conditions (e.g., HIV, diabetes, hypertension, cardiovascular disease, chronic bronchitis/chronic obstructive pulmonary disease (COPD), asthma) and relevant exposures will be determined.

# Data analysis and statistical considerations

## Variables of interest

Exposures of interest will include age, sex, occupation, food insecurity, alcohol and tobacco consumption and history of TB and other conditions. Measures of socio-economic position will be included where available (for ERASE-TB, asset score and household income). These are all factors which are associated with increased risk of TB, and of chronic conditions of interest.

Outcomes of interest are TB and chronic diseases.

The chronic conditions of interest are: hypertension, diabetes, poorly reversible obstructive airways disease, under or overweight, anaemia and HIV. Where assessment has been conducted at multiple timepoints (for example, blood pressure is assessed at each ERASE-TB study visit) the first assessment will be used to define presence/absence of disease. These will be measured and categorized as defined in the study protocol (Table 1).

*Table 1: Definitions for chronic conditions*

| Condition | Definition |
|---|---|
| **HIV** | a) self-report of previous positive HIV test, or<br>b) self-report of being on ART, or<br>c) positive HIV test result |
| **Previous TB** | Self-report of previous TB episode |
| **TB** | Microbiologically confirmed (Xpert Ultra positive) or clinical diagnosis of TB |
| **Anaemia** | Haemoglobin concentration compatible with any degree of anaemia as shown in supplementary table 4 |
| **Chronic lung disease** | a) Self report of chronic lung disease (asthma, COPD or silicosis)<br>b) Obstructive or mixed defect on pre-bronchodilator spirometry (less than lower limit of normal using Global Lung Initiative 'Other/Mixed' ethnicity reference standard) |
| **Hypertension** | a) self-reported diagnosis of hypertension, or<br>b) self-report of being on anti-hypertensive medications, or<br>c) systolic BP ≥140mmHg, or<br>d) diastolic BP ≥90mmHg.[4] |
| **Diabetes** | a) self-reported diagnosis of diabetes or<br>b) self-report of being on anti-diabetic medications, or<br>c) HbA1c ≥6.5%.[5] |
| **Underweight** | BMI for age Z score <-1 among adolescents (<19 years) and BMI <18.5kg/m2 among adults (≥19 years). |

**Abbreviations**: ART = anti-retroviral therapy, BMI = body mass index; BP = blood pressure; COPD = chronic obstructive pulmonary disease

*Table 2: BMI category definitions as per WHO*

| Category | Adolescent definition (<19 years)<br>BMI for age Z score thresholds | Adult definition (≥19 years)<br>Absolute BMI thresholds |
|---|---|---|
| Moderate/severe underweight | <-2 | <17 kg/m2 |
| Mild underweight | ≥-2 & <-1 | 17-18.4 kg/m2 |
| Normal weight | ≥-1 & ≤+1 | 18.5-24.9 kg/m2 |
| Overweight | >+1 & ≤+2 | 25-29.9 kg/m2 |

| Category | Adolescent definition (<19 years) BMI for age Z score thresholds | Adult definition (≥19 years) Absolute BMI thresholds |
|---|---|---|
| Obese | >+2 | ≥30 kg/m2 |

*Table 3: WHO classification of anaemia based on haemoglobin concentration (g/L)*

| Population | | Non-anaemia | Mild anaemia | Moderate anaemia | Severe anaemia |
|---|---|---|---|---|---|
| 10-11 years | | ≥115 | 110-114 | 80-109 | <70 |
| 12-14 years | | ≥120 | 110-119 | 80-109 | <80 |
| ≥15 years | Non-pregnant women | ≥120 | 110-119 | 80-109 | <80 |
| | Pregnant women | ≥110 | 100-109 | 70-99 | <70 |
| | Men | ≥130 | 110-129 | 80-109 | <80 |

Multimorbidity will be defined as two or more conditions.[3] In addition, chronic diseases and will be considered as being controlled or uncontrolled (Table 4), since achieving disease control averts the adverse health consequences of disease (for example, viral suppression with ART, or blood pressure within the normal range on anti-hypertensive medication).

*Table 4: Definitions of controlled and uncontrolled disease for chronic conditions of interest*

| | Controlled disease | Uncontrolled disease |
|---|---|---|
| **Diabetes**<br>**Hypertension**<br>**Anaemia** | Participant has the condition (self-report, evidence of being on treatment, or a previous positive screening result) but does not screen positive. | Participant screens positive for the condition; either as a new diagnosis or on the background of previously diagnosed disease (self-report, evidence of being on treatment, or a previous positive screening result). |
| **HIV** | Participant reports that they have the condition but that they are on ART (and CD4 count >350 cells/uL, if available) | Participant tests positive for HIV and is not known to have HIV, is not currently on ART or has low CD4 count. |
| **TB** | Participant reports a previous history of TB (or has a previous positive test result) and is currently on TB treatment | Participant tests positive for TB |

## Statistical methods

Cross-sectional analyses to describe the prevalence of chronic conditions will primarily be descriptive, calculating the prevalence of each condition, together with 95% confidence intervals. Confidence intervals will be adjusted for household level clustering using appropriate methods. Given each site contributes equally to the dataset (n=700 in each), we will not account for site-level clustering in the analyses. Key subgroups of interest are those stratified

by: gender, age and study site. We will additionally use direct age standardization to standardize prevalence estimates to the WHO reference population, facilitating comparisons with national level estimates and across sites (and, in future, across studies).

In ERASE-TB, analyses will be conducted both using the individual as the unit of analysis and at the household-level. For the latter, we will fit a multi-level random effects model to estimate the degree of clustering (as an intra-cluster correlation coefficient) of each outcome of interest and explore whether the clustering is explained by individual and household-level exposures by adjusting for these in the model. To illustrate: clustering of diabetes within households may be observed in univariable analysis but not after adjustment for age, suggesting the clustering was a result of individuals in households being more similar to each other, than to people in a different household, in terms of age.[18]

## Sample size

This study will include all participants in ERASE-TB (n=2100).
