# Supplementary material for: HIV, malnutrition, and noncommunicable disease epidemics among tuberculosis-affected households in east and southern Africa: A cross-sectional analysis of the ERASE-TB cohort
Source: PLoS Med. 2024 Sep 16;21(9):e1004452. doi: 10.1371/journal.pmed.1004452 (PMC11441706; doi:10.1371/journal.pmed.1004452)
Supplement: S1 Appendix — (PDF) [file pmed.1004452.s004.pdf]

HIV, malnutrition, and non-communicable disease epidemics among tuberculosis-affected households in east and southern Africa: a cross-sectional analysis of the ERASE-TB cohort

Supplementary materials

CONTENTS

Supplementary methods *3*
Definition of household contacts 3
Testing for NCDs in ERASE-TB 3
Table A: Timing of tests for chronic conditions among participants included in analysis, stratified by site (N = 1940) 3
Definitions for chronic conditions 4
Table C: BMI categories used in this study. 5
Table D: Classification of anaemia based on haemoglobin concentration (g/L) used in this study 5
Definitions of other individual-level exposures 5
Definitions of household-level exposures 5
Packages used for analysis 5
Table E: Estimates of chronic condition prevalence from population-representative surveys, and estimates of associated increased risk of TB 6

Supplementary results *7*
Table F: Missing data for tests for chronic conditions in study population, stratified by site (N = 2109) 7
Table G: Characteristics of study participants included and excluded from analysis dataset, stratified by study site (N = 2109) 8
Table H (part A): Participant characteristics stratified by sex (N = 1958) 10
Table H (Part B): Participant characteristics stratified by site and sex (N = 1958) 11
Figure A: Age distribution of household contacts, relative to the index case (N = 1958) 12
Table I: Chronic conditions among TB household contacts, stratified by sex and age category, including confidence intervals (N = 1958) 13
Table J: Chronic conditions among TB household contacts, stratified by site (N = 1958) 15
Figure B: Prevalence of chronic conditions among TB household contacts stratified by age category and sex (N = 1958) 16
Figure C: Prevalence of chronic conditions among TB household contacts stratified by age category, sex and study site (N = 1958) 17
Table K: Site specific age-standardised prevalence of chronic conditions 18
Figure D: Association between HbA1c, systolic BP and diastolic BP, and age 19
Figure E: Association between HbA1c, systolic and diastolic BP and BMI category 20
Figure F: BMI distribution by age 21
Table M: Cascade of care for HIV, diabetes and hypertension 22
Table N: Prevalence of chronic conditions at household level (N=786 households) 23
Figure H: Overlap of underweight, stunting and overweight/obesity at household level (N = 786 households) 23

Additional tables added at peer review *24*
Table O: Demographic characteristics of TB household contacts in Tanzania, stratified by residence area (N = 649*) 24
Table P: Chronic conditions among TB household contacts contacts in Tanzania, stratified by residence area (N = 649*) 25

Supplementary methods

Definition of household contacts

People were eligible to participate in the study if they were aged ≥10 years and living in the same household as someone who had recently (within the past four weeks) been diagnosed with tuberculosis. Living in the same household was defined as spending at least thrre nights per week in the same household as the person with TB.

Testing for NCDs in ERASE-TB

Due to logistical and regulatory requirements, HbA1c, Hb and blood pressure testing were not available at study initiation and were introduced whilst data collection was ongoing. Where participants were not tested at baseline, we used results obtained at follow up visits (Supplementary table 2). There was no evidence that the distribution of these variables differed between people tested at baseline and those tested during follow up (data not shown).

Table A: Timing of tests for chronic conditions among participants included in analysis, stratified by site (N = 1940)

Characteristic	Overall<br>N = 1,958	Mozambique<br>N = 680	Tanzania<br>N = 650	Zimbabwe<br>N = 628
**HIV visit**				
Visit-1	1,690 (97%)	581 (98%)	592 (97%)	517 (97%)
Visit-2	13 (0.7%)	1 (0.2%)	9 (1.5%)	3 (0.6%)
Visit-3-5	32 (1.8%)	8 (1.4%)	9 (1.5%)	15 (2.8%)
**HIV CD4 visit**				
Visit-1	240 (81%)	88 (84%)	74 (83%)	78 (76%)
Visit-2	30 (10%)	13 (12%)	10 (11%)	7 (6.9%)
Visit-3-5	26 (8.8%)	4 (3.8%)	5 (5.6%)	17 (17%)
**HbA1c visit**				
Visit-1	1,155 (59%)	453 (67%)	316 (49%)	386 (62%)
Visit-2	507 (26%)	180 (27%)	166 (26%)	161 (26%)
Visit-3-5	284 (15%)	42 (6.2%)	168 (26%)	74 (12%)
**BP visit**				
Visit-1	1,423 (73%)	603 (90%)	403 (63%)	417 (66%)
Visit-2	358 (18%)	31 (4.6%)	154 (24%)	173 (28%)
Visit-3-5	159 (8.2%)	37 (5.5%)	84 (13%)	38 (6.1%)
**Weight visit**				
Visit-1	1,951 (100%)	674 (99%)	649 (100%)	628 (100%)
Visit-2	7 (0.4%)	6 (0.9%)	1 (0.2%)	0 (0%)
**Hb visit**				
Visit-1	1,381 (71%)	661 (97%)	318 (49%)	402 (64%)
Visit-2	336 (17%)	2 (0.3%)	164 (25%)	170 (27%)
Visit-3-5	241 (12%)	17 (2.5%)	168 (26%)	56 (8.9%)

Footnotes: presented as number (percentage).

Definitions for chronic conditions

Definitions for the chronic conditions included in this analysis are shown in supplementary table 2.

For chronic lung disease, the Global Lung Initiative 'Other/mixed' reference standard was used as previously recommended for South Africa.[1] Given a restrictive pattern of spirometry is insufficient for a diagnosis of restrictive lung disease without further evaluation, we did not include this as a criterion for chronic lung disease in this study.[2]

Combined BMI categories were created across both adolescents (here defined as <19 years) and adults, using the terminology usually applied for adult BMI, as shown in supplementary table 3. This approach was selected to aid comparison across all ages included in this study as the BMI for age thresholds use approximate to the adult cut-offs for an individual aged 19.

BMI for age Z scores were calculated by using the WHO reference standard for children and adolescents, and applying the highest reference stratum (that for 19 year olds) to all adults in the study population.

Stunting was only calculated among adolescents. Mild stunting was defined as a height of age Z score of <-1 and ≥-2; moderate stunting as Z score of <-2 and ≥-3 and severe stunting as Z score <-3.

Anaemia was categorised using WHO-recommended thresholds, as shown in supplementary table 4.[3]

Blood pressure was categorised according to WHO (all units mmHg): normal, SBP <130 / DBP<85; high-normal BP, SBP 130-139 / DBP 85-89; Grade 1 hypertension, SBP 140-159 / DBP 90-99"; Grade 2 hypertension, SBP ≥160 / DBP ≥100.

Table B: Definitions for chronic conditions

Condition	Definition	Definition of controlled disease
**HIV**	a) self-report of previous positive HIV test, or<br>b) self-report of being on ART, or<br>c) positive HIV test result	Known HIV on ART and CD4 count ≤350cells/uL
**Previous TB**	Self-report of previous TB episode	NA
**TB**	Microbiologically confirmed (Xpert Ultra positive) or clinical diagnosis of TB	TB currently on treatment or completed treatment with successful outcome
**Anaemia**	Haemoglobin concentration compatible with any degree of anaemia as shown in supplementary table 4	NA
**Chronic lung disease**	a) Self report of chronic lung disease (asthma, COPD or silicosis)<br>b) Obstructive or mixed defect on pre-bronchodilator spirometry (less than lower limit of normal using Global Lung Initiative 'Other/Mixed' ethnicity reference standard)	NA
**Hypertension**	a) self-reported diagnosis of hypertension, or<br>b) self-report of being on anti-hypertensive medications, or<br>c) systolic BP ≥140mmHg, or<br>d) diastolic BP ≥90mmHg.[4]	Known hypertension and systolic BP>140mmHg and diastolic BP < 90mmHg
**Diabetes**	a) self-reported diagnosis of diabetes or<br>b) self-report of being on anti-diabetic medications, or<br>c) HbA1c ≥6.5%.[5]	Known diabetes with HbA1c < 6.5%
**Underweight**	BMI for age Z score <-1 among adolescents (<19 years) and BMI <18.5kg/m2 among adults (≥19 years).	NA

Abbreviations: ART = anti-retroviral therapy, BMI = body mass index; BP = blood pressure; COPD = chronic obstructive pulmonary disease; NA = not assessed

Table C: BMI categories used in this study.

Category	Adolescent definition (<19 years) BMI for age Z score thresholds	Adult definition (≥19 years) Absolute BMI thresholds
Moderate/severe underweight	<-2	<17 kg/m2
Mild underweight	≥-2 & <-1	17-18.4 kg/m2
Normal weight	≥-1 & ≤+1	18.5-24.9 kg/m2
Overweight	>+1 & ≤+2	25-29.9 kg/m2
Obese	>+2	≥30 kg/m2

Table D: Classification of anaemia based on haemoglobin concentration (g/L) used in this study

Population		Non-anaemia	Mild anaemia	Moderate anaemia	Severe anaemia
10-11 years		≥115	110-114	80-109	<70
12-14 years		≥120	110-119	80-109	<80
≥15 years	Non-pregnant women	≥120	110-119	80-109	<80
	Pregnant women	≥110	100-109	70-99	<70
	Men	≥130	110-129	80-109	<80

Definitions of other individual-level exposures

AUDIT-C (Alcohol Use Disorder Identification Test) is a three-question screening tool that quantifies alcohol misuse. Higher scores correlate with greater severity of alcohol misuse. It was categorised as per the original authors (scores ≥4 among men and ≥3 among women suggest alcohol misuse).[6]

Crowding was defined as per the UN Habitat: ≥3 per people per habitable room.[7]

Food insecurity was self-report of having had insufficient food in the past 6 months.

Definitions of household-level exposures

A threshold of 1.90 United States Dollars (USD) per person per day was used to define poverty in this study this was the international poverty line threshold in 2021 when data collection began. We note that in September 2022 the threshold was increased to 2.15USD (https://pip.worldbank.org).

Packages used for analysis

- Calculation of prevalence estimates, including age-standardisation: survey v4.2-1.
- Creation of intersection plots: ComplexUpset v1.3.3.

Table E: Estimates of chronic condition prevalence from population-representative surveys, and estimates of associated increased risk of TB

	Mozambique	Tanzania	Zimbabwe	Risk of TB
**HIV***	12.5%[8] M 9.5% / F 15.0%<br>Maputo: M 9.8% / F 18.6%	5%[9] M 3.4% / F 6.3%<br>Mbeya M 4.9% / F 13.2%	12%[10] M 10.2% / F 15.3%<br>Harare: M 10.5% / F 14.2%	RR 18[11]
**Diabetes†**	2.4% /<br>Age-st: 3.3%[12]	10.3%<br>Age-st: 12.3%[12]	1.5%<br>Age-st: 2.1%[12]	OR 2.3[17]
**Hypertension‡**	34.3% /<br>Age-st: 38.6%[13]	30.9% /<br>Age-st: 33.2%[13]	37.6% Age-st: 42.3%[13]	NR
**Underweight§**	C&A:<br>M 4.2% / F 2.8%<br>Adults: M 12.0% / F 9.9%[14]	C&A:<br>M 9.0% / F 4.1%<br>Adults:<br>M 12.3% / F 9.8%[14]	C&A:<br>M 9.1% / F 1.9%[14]<br>Adults:<br>M 11.1% / F 4.9%	OR 4.96[15]
**Overweight + obesity§**	C&A:<br>M 9.1% / F 20.0%<br>Adults:<br>M 21.3% / F 44.1%[14]	C&A:<br>M 9.3% / F 19.3%<br>Adults:<br>M 23.6% / F 48.2%[14]	C&A:<br>M 7.9% / F 28.5%<br>Adults:<br>M 26.9% / F 78.1%[14]	OR 0.26[15]
**Anaemia§**	F 47.9%[14]	F 38.9%[14]	28.9%[14]	OR 2.01[16]

Footnotes: Data are from: * population-representative HIV prevalence surveys including adults aged 15 and older. † International Diabetes Federation estimates for adults 20, 79 years.[19] ‡ World Health Organization Global Health Observatory data for adults aged 30, 79 years. § Global Nutrition Report for Children and adolescents 5-19 years (C&A) and all adults, respectively. For both, data are stratified by sex (M = male, F = female) Anaemia estimates only reported among females. ll The World Health Organization indicator for alcohol is heavy episodic drinking (six or more drinks on one occasion in the past 30 days. The estimate of increased risk of TB uses 'hazardous alcohol use' (as defined by authors of included studies in the systematic review). **Abbreviations:** Age-st = age-standardised; RR = risk ratio; OR = odds ratio

Supplementary results

Table F: Missing data for tests for chronic conditions in study population, stratified by site (N = 2109)

Characteristic	**Overall**, N = 2,109	**Mozambique**, N = 710	**Tanzania**, N = 699	**Zimbabwe**, N=700
**HIV test done***				
No	0 (0%)	0 (0%)	0 (0%)	0 (0%)
Yes	1,817 (86%)	601 (85%)	620 (89%)	596 (85%)
Not applicable	292 (14%)	109 (15%)	79 (11%)	104 (15%)
**CD4 count done**†				
No	23 (1.1%)	9 (1.3%)	8 (1.1%)	6 (0.9%)
Yes	304 (14%)	109 (15%)	87 (12%)	108 (16%)
Not applicable	1,771 (84%)	591 (83%)	603 (86%)	577 (84%)
Unknown	11	1	1	9
**Spiro done**‡				
No	434 (21%)	157 (22%)	118 (17%)	159 (23%)
Yes	1,675 (79%)	553 (78%)	581 (83%)	541 (77%)
**HbA1c done§**				
No	76 (3.6%)	21 (3.0%)	10 (1.4%)	45 (6.4%)
Yes	1,412 (67%)	466 (66%)	485 (69%)	461 (66%)
Not applicable	621 (29%)	223 (31%)	204 (29%)	194 (28%)
**BP done§**				
No	37 (1.8%)	1 (0.1%)	36 (5.2%)	0 (0%)
Yes	1,451 (69%)	486 (68%)	459 (66%)	506 (72%)
Not applicable	621 (29%)	223 (31%)	204 (29%)	194 (28%)
**BMI done**				
No	2 (<0.1%)	0 (0%)	1 (0.1%)	1 (0.1%)
Yes	2,107 (100%)	710 (100%)	698 (100%)	699 (100%)
**Hb done**				
No	56 (2.7%)	7 (1.0%)	2 (0.3%)	47 (6.7%)
Yes	2,053 (97%)	703 (99%)	697 (100%)	653 (93%)

Footnotes: Presented as number (percentage). * HIV test is not applicable if the person self-reported that they were living with HIV. † CD4 is unknown where the HIV status is missing, therefore whether a CD4 count is needed is missing. ‡ Spiro done means that there is a spirometry result meeting ERS/ATS quality criteria available. § HbA1c and BP are not applicable if the person is under the age of 18 years at recruitment.

Table G: Characteristics of study participants included and excluded from analysis dataset, stratified by study site (N = 2109)

Characteristic	**Mozambique**, N = 710		**Tanzania**, N = 699		**Zimbabwe**, N = 700	
	**Excluded** N = 29	**Included** N = 681	**Excluded** N = 49	**Included** N = 650	**Excluded** N = 61	**Included** N = 639
**Sex**						
Female	17 (59%)	412 (60%)	31 (63%)	416 (64%)	39 (64%)	397 (62%)
Male	12 (41%)	269 (40%)	18 (37%)	234 (36%)	22 (36%)	242 (38%)
**Age, years**	30 (23–36)	23 (16–39)	29 (22–38)	29 (16–44)	25 (18–36)	28 (17–42)
**Age category**						
10-17 years	1 (3.4%)	222 (33%)	1 (2.0%)	203 (31%)	13 (21%)	181 (28%)
18-39 years	21 (72%)	298 (44%)	38 (78%)	235 (36%)	40 (66%)	280 (44%)
40+ years	7 (24%)	161 (24%)	10 (20%)	212 (33%)	8 (13%)	178 (28%)
**Highest educational level***						
None or primary school	12 (46%)	254 (39%)	38 (83%)	472 (75%)	11 (21%)	175 (29%)
At least secondary school	14 (54%)	390 (61%)	8 (17%)	156 (25%)	41 (79%)	433 (71%)
**Pregnant†**						
No	17 (100%)	404 (98%)	29 (94%)	402 (97%)	37 (95%)	380 (96%)
Yes	0 (0%)	8 (1.9%)	2 (6.5%)	14 (3.4%)	2 (5.1%)	17 (4.3%)
**Smoking status**						
Non smoker	26 (90%)	655 (96%)	44 (90%)	612 (94%)	50 (82%)	563 (88%)
Smoker (current and/or former)	3 (10%)	26 (3.8%)	5 (10%)	38 (5.8%)	11 (18%)	75 (12%)
**Pack years smoking**	3.2 (2.2–8.3)	1.8 (1.2–3.6)	2.0 (1.5–3.3)	1.5 (0.5–4.2)	0.3 (0.2–1.9)	2.0 (0.7–5.6)
**Alcohol consumption**						
Never drunk alcohol	15 (52%)	438 (64%)	31 (65%)	425 (65%)	47 (78%)	453 (71%)
Alcohol, AUDIT-C negative	3 (10%)	162 (24%)	11 (23%)	124 (19%)	8 (13%)	99 (15%)
Alcohol, AUDIT-C positive	11 (38%)	80 (12%)	6 (13%)	101 (16%)	5 (8.3%)	87 (14%)
**Insufficient food** ‡						
No	18 (64%)	439 (65%)	47 (96%)	612 (94%)	40 (66%)	379 (59%)
Yes	10 (36%)	241 (35%)	2 (4.1%)	38 (5.8%)	21 (34%)	260 (41%)
**Known HIV**						
No	18 (62%)	583 (86%)	40 (82%)	580 (89%)	53 (87%)	543 (85%)
Yes	11 (38%)	98 (14%)	9 (18%)	70 (11%)	8 (13%)	96 (15%)
**On ART**						
No	1 (9.1%)	4 (4.1%)	0 (0%)	2 (2.9%)	1 (13%)	6 (6.3%)
Yes	10 (91%)	93 (96%)	9 (100%)	67 (97%)	7 (88%)	89 (94%)
**Known diabetes**						
No	29 (100%)	669 (98%)	49 (100%)	644 (99%)	61 (100%)	628 (98%)
Yes	0 (0%)	12 (1.8%)	0 (0%)	6 (0.9%)	0 (0%)	11 (1.7%)
**Known hypertension**						
No	26 (90%)	622 (91%)	49 (100%)	628 (97%)	57 (93%)	573 (90%)
Yes	3 (10%)	59 (8.7%)	0 (0%)	22 (3.4%)	4 (6.6%)	66 (10%)
**Any lung disease**						
No	25 (89%)	630 (93%)	20 (69%)	496 (85%)	51 (94%)	602 (97%)
Yes	3 (11%)	47 (6.9%)	9 (31%)	89 (15%)	3 (5.6%)	20 (3.2%)
**Asthma**						
No	27 (96%)	640 (94%)	26 (96%)	582 (98%)	53 (96%)	617 (98%)
Yes	1 (3.6%)	38 (5.6%)	1 (3.7%)	13 (2.2%)	2 (3.6%)	12 (1.9%)
**Pneumonia**						
No	28 (100%)	678 (100%)	39 (91%)	532 (89%)	53 (98%)	628 (100%)
Yes	0 (0%)	0 (0%)	4 (9.3%)	66 (11%)	1 (1.9%)	2 (0.3%)
**Previous TB**						
No	26 (90%)	622 (91%)	47 (96%)	625 (96%)	59 (97%)	602 (94%)
Yes	3 (10%)	59 (8.7%)	2 (4.1%)	25 (3.8%)	2 (3.3%)	37 (5.8%)
**Relationship to index case**						
Spouse	10 (34%)	109 (16%)	14 (29%)	123 (19%)	18 (30%)	99 (15%)

	Mozambique, N = 710		Tanzania, N = 699		Zimbabwe, N = 700	
**Characteristic**	**Excluded** N = 29	**Included** N = 681	**Excluded** N = 49	**Included** N = 650	**Excluded** N = 61	**Included** N = 639
Parent	3 (10%)	193 (28%)	11 (22%)	165 (25%)	13 (21%)	144 (23%)
Sibling	8 (28%)	134 (20%)	14 (29%)	118 (18%)	12 (20%)	119 (19%)
Child	4 (14%)	90 (13%)	1 (2.0%)	91 (14%)	7 (11%)	88 (14%)
Other	4 (14%)	155 (23%)	9 (18%)	153 (24%)	11 (18%)	189 (30%)

Footnotes: Presented as number (percentage) or median (interquartile range). *Educational level not known for 88 participants. †The denominator for pregnancy is the number of women. ‡ Insufficient food was defined as participants as answering yes to ‘was there any day in the past six months where you did not have enough food’.
Abbreviations: AUDIT-C = alcohol use identification test, short form; TB = tuberculosis; N = number.

Table H (part A): Participant characteristics stratified by sex (N = 1958)

Characteristic	**Overall**, N = 1,958	**Women**, N = 1,217	**Men**, N = 741
**Site**			
Mozambique	680 (35%)	411 (34%)	269 (36%)
Tanzania	650 (33%)	416 (34%)	234 (32%)
Zimbabwe	628 (32%)	390 (32%)	238 (32%)
**Age, years**	27 (16, 42)	30 (18, 45)	21 (14, 37)
**Age category**			
10-17 years	602 (31%)	305 (25%)	297 (40%)
18-39 years	807 (41%)	525 (43%)	282 (38%)
40+ years	549 (28%)	387 (32%)	162 (22%)
**Highest educational level***			
None or primary school	897 (48%)	583 (50%)	314 (45%)
At least secondary school	973 (52%)	587 (50%)	386 (55%)
Unknown	88	47	41
**Pregnant†**	38 (3.1%)	38 (3.1%)	0 (NA%)
Unknown	741	0	741
**Smoking status**			
Non smoker	1,819 (93%)	1,200 (99%)	619 (84%)
Smoker (current and/or former)	138 (7.1%)	17 (1.4%)	121 (16%)
Unknown	1	0	1
**Pack years smoking**	1.8 (0.6, 4.6)	2.0 (0.5, 3.5)	1.8 (0.6, 5.0)
Unknown	1,829	1,201	628
**Alcohol consumption**			
Never drunk alcohol	1,308 (67%)	866 (71%)	442 (60%)
Alcohol, AUDIT-C negative	384 (20%)	240 (20%)	144 (19%)
Alcohol, AUDIT-C positive	265 (14%)	110 (9.0%)	155 (21%)
Unknown	1	1	0
**Insufficient food‡**	535 (27%)	351 (29%)	184 (25%)
Unknown	1	0	1
**Relationship to index case**			
Spouse	330 (17%)	267 (22%)	63 (8.5%)
Parent	499 (25%)	284 (23%)	215 (29%)
Sibling	368 (19%)	184 (15%)	184 (25%)
Child	267 (14%)	193 (16%)	74 (10.0%)
Other	494 (25%)	289 (24%)	205 (28%)

Footnotes: Presented as number (percentage) or median (interquartile range). *Educational level not known for 88 participants. † The denominator for pregnancy is the number of women. ‡ Insufficient food was defined as participants as answering yes to 'was there any day in the past six months where you did not have enough food'. **Abbreviations**: AUDIT-C = alcohol use identification test, short form; TB = tuberculosis; N = number.

Table H (Part B): Participant characteristics stratified by site and sex (N = 1958)

	Mozambique, N = 680		Tanzania, N = 650		Zimbabwe, N = 628	
**Characteristic**	**Women**, N = 411	**Men**, N = 269	**Women**, N = 416	**Men**, N = 234	**Women**, N = 390	**Men**, N = 238
**Age, years**	27 (18, 45)	19 (14, 31)	34 (18, 47)	22 (14, 40)	29 (18, 42)	23 (15, 41)
**Age category**						
10-17 years	107 (26%)	114 (42%)	107 (26%)	96 (41%)	91 (23%)	87 (37%)
18-39 years	184 (45%)	114 (42%)	155 (37%)	80 (34%)	186 (48%)	88 (37%)
40+ years	120 (29%)	41 (15%)	154 (37%)	58 (25%)	113 (29%)	63 (26%)
**Highest educational level***						
None or primary school	166 (42%)	87 (35%)	308 (77%)	164 (73%)	109 (29%)	63 (28%)
At least secondary school	227 (58%)	163 (65%)	94 (23%)	62 (27%)	266 (71%)	161 (72%)
**Pregnant†**	8 (1.9%)	0 (NA%)	14 (3.4%)	0 (NA%)	16 (4.1%)	0 (NA%)
**Smoking status**						
Non smoker	407 (99%)	247 (92%)	411 (99%)	201 (86%)	382 (98%)	171 (72%)
Smoker (current and/or former)	4 (1.0%)	22 (8.2%)	5 (1.2%)	33 (14%)	8 (2.1%)	66 (28%)
**Pack years smoking**	3.7 (2.9, 5.7)	1.6 (1.1, 2.8)	2.5 (0.5, 2.5)	1.5 (0.5, 4.3)	1 (0, 2)	3 (1, 6)
**Alcohol consumption**						
Never drunk alcohol	271 (66%)	166 (62%)	277 (67%)	148 (63%)	318 (82%)	128 (54%)
Alcohol, AUDIT-C negative	110 (27%)	52 (19%)	78 (19%)	46 (20%)	52 (13%)	46 (19%)
Alcohol, AUDIT-C positive	29 (7.1%)	51 (19%)	61 (15%)	40 (17%)	20 (5.1%)	64 (27%)
**Insufficient food‡**	156 (38%)	85 (32%)	24 (5.8%)	14 (6.0%)	171 (44%)	85 (36%)
**Relationship to index case**						
Spouse	85 (21%)	24 (8.9%)	102 (25%)	21 (9.0%)	80 (21%)	18 (7.6%)
Parent	98 (24%)	95 (35%)	104 (25%)	61 (26%)	82 (21%)	59 (25%)
Sibling	66 (16%)	68 (25%)	64 (15%)	54 (23%)	54 (14%)	62 (26%)
Child	70 (17%)	20 (7.4%)	61 (15%)	30 (13%)	62 (16%)	24 (10%)
Other	92 (22%)	62 (23%)	85 (20%)	68 (29%)	112 (29%)	75 (32%)

Footnotes: Presented as number (percentage) or median (interquartile range). *Educational level not known for 88 participants. † The denominator for pregnancy is the number of women. ‡ Insufficient food was defined as participants as answering yes to 'was there any day in the past six months where you did not have enough food'.

Figure A: Age distribution of household contacts, relative to the index case (N = 1958)

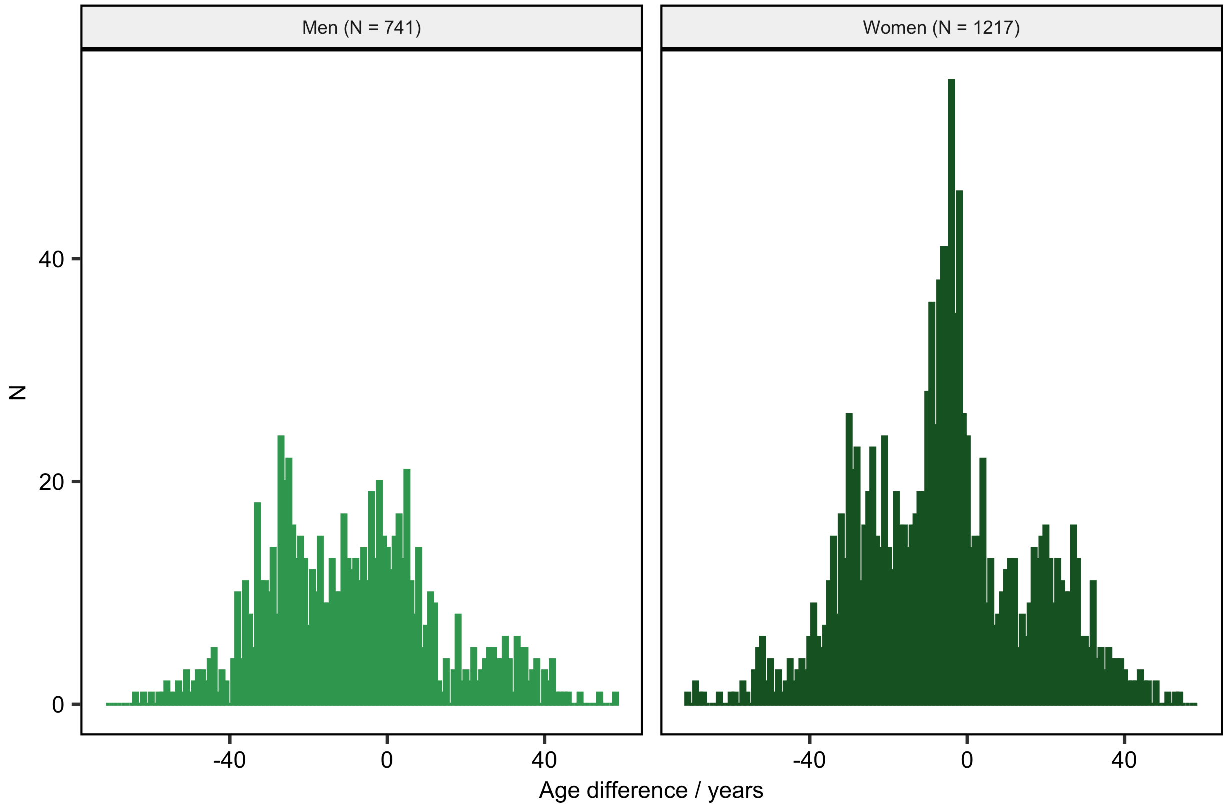

Abbreviations: N = number

Table I: Chronic conditions among TB household contacts, stratified by sex and age category, including confidence intervals (N = 1958)

Condition	Level	Overall	Women	Men	10-17 years	18-39 years	40+ years
**All participants**		**N = 1958**	**N = 1217**	**N = 741**	**N = 602**	**N = 807**	**N = 549**
HIV		15.0% (13.2%, 17.0%)	19.1% (16.7%, 21.6%)	8.4% (6.4%, 10.8%)	2.7% (1.6%, 4.3%)	14.3% (11.9%, 17.0%)	29.7% (25.6%, 34.1%)
CD4 category	≥500cells/uL	63.5% (57.7%, 68.9%)	66.7% (60.2%, 72.6%)	51.6% (39.1%, 64.0%)	76.5% (51.3%, 90.9%)	59.6% (50.3%, 68.4%)	64.8% (57.0%, 72.0%)
	200-499cells/uL	32.1% (27.0%, 37.7%)	29.9% (24.3%, 36.2%)	40.3% (28.8%, 53.0%)	23.5% (9.1%, 48.7%)	36.8% (28.4%, 46.2%)	29.7% (23.1%, 37.2%)
	<200cells/uL	4.4% (2.6%, 7.4%)	3.4% (1.7%, 6.7%)	8.1% (3.6%, 17.2%)		3.5% (1.3%, 9.0%)	5.5% (2.9%, 10.0%)
CD4 (cells / uL)		584.0 (546.0, 616.0)	603.0 (561.0, 643.0)	526.0 (408.0, 606.0)	678.0 (515.0, 1060.0)	565.0 (500.0, 656.0)	589.0 (536.0, 616.0)
BMI category	Moderate/severe underweight	3.9% (3.0%, 4.9%)	2.5% (1.8%, 3.7%)	6.1% (4.5%, 8.2%)	8.1% (6.1%, 10.8%)	2.2% (1.4%, 3.5%)	1.6% (0.9%, 3.1%)
	Mild underweight	13.5% (12.0%, 15.2%)	9.6% (8.0%, 11.5%)	20.0% (17.1%, 23.2%)	27.4% (23.8%, 31.4%)	9.2% (7.3%, 11.4%)	4.7% (3.2%, 6.9%)
	Healthy weight	54.5% (52.2%, 56.9%)	49.8% (46.9%, 52.7%)	62.3% (58.6%, 66.0%)	57.0% (52.7%, 61.1%)	59.4% (55.6%, 63.0%)	44.8% (40.8%, 48.9%)
	Overweight	17.4% (15.7%, 19.2%)	22.0% (19.7%, 24.5%)	9.7% (7.8%, 12.1%)	6.3% (4.6%, 8.6%)	18.8% (16.2%, 21.7%)	27.3% (23.7%, 31.2%)
	Obese	10.7% (9.4%, 12.2%)	16.0% (14.0%, 18.2%)	1.9% (1.1%, 3.2%)	1.2% (0.6%, 2.4%)	10.4% (8.4%, 12.8%)	21.5% (18.4%, 25.0%)
Chronic lung disease		10.3% (8.9%, 11.9%)	11.0% (9.2%, 13.1%)	9.2% (7.3%, 11.7%)	9.1% (6.9%, 11.9%)	9.4% (7.5%, 11.8%)	13.0% (10.3%, 16.2%)
Anaemia		17.7% (15.9%, 19.7%)	20.7% (18.3%, 23.3%)	12.8% (10.4%, 15.6%)	16.6% (13.6%, 20.1%)	18.0% (15.4%, 20.8%)	18.6% (15.4%, 22.2%)
Anaemia category	None	82.3% (80.3%, 84.1%)	79.3% (76.7%, 81.7%)	87.2% (84.4%, 89.6%)	83.4% (79.9%, 86.4%)	82.0% (79.2%, 84.6%)	81.4% (77.8%, 84.6%)
	Mild anaemia	11.5% (10.1%, 13.1%)	12.2% (10.5%, 14.3%)	10.3% (8.2%, 12.8%)	11.8% (9.3%, 14.8%)	10.7% (8.7%, 13.0%)	12.4% (9.8%, 15.5%)
	Moderate anaemia	5.6% (4.6%, 6.9%)	7.6% (6.2%, 9.2%)	2.4% (1.5%, 3.9%)	4.3% (2.8%, 6.7%)	6.6% (5.1%, 8.4%)	5.6% (4.0%, 8.0%)
	Severe anaemia	0.6% (0.3%, 1.2%)	0.9% (0.4%, 1.9%)	0.1% (0.0%, 1.0%)	0.5% (0.2%, 1.5%)	0.7% (0.3%, 1.9%)	0.5% (0.2%, 1.7%)
Hb (g/dL)		135.0 (134.0, 136.0)	130.0 (129.0, 132.0)	144.0 (142.0, 146.0)		137.0 (136.0, 139.0)	135.0 (133.0, 138.0)
**Adolescents**		**N = 602**	**N = 305**	**N = 297**	**N = 602**		
Stunting	Normal	56.6% (52.2%, 61.0%)	62.3% (56.4%, 67.9%)	50.8% (44.6%, 57.0%)	56.6% (52.2%, 61.0%)		
	Mild stunting	26.7% (23.5%, 30.3%)	26.2% (21.8%, 31.3%)	27.3% (22.4%, 32.8%)	26.7% (23.5%, 30.3%)		
	Moderate stunting	12.8% (10.2%, 15.9%)	8.9% (6.2%, 12.5%)	16.8% (12.7%, 22.0%)	12.8% (10.2%, 15.9%)		
	Severe stunting	3.8% (2.5%, 5.8%)	2.6% (1.3%, 5.1%)	5.1% (3.1%, 8.2%)	3.8% (2.5%, 5.8%)		
**Adults**		**N = 1356**	**N = 912**	**N = 444**		**N = 807**	**N = 549**
Diabetes		9.4% (7.9%, 11.1%)	10.4% (8.6%, 12.6%)	7.2% (5.2%, 9.9%)		4.8% (3.5%, 6.6%)	16.0% (13.1%, 19.5%)
HbA1c category	<6.0%	62.8% (59.8%, 65.6%)	62.5% (59.1%, 65.8%)	63.3% (58.4%, 67.9%)		68.9% (65.3%, 72.2%)	53.7% (49.2%, 58.2%)
	6.0-6.4%	28.8% (26.2%, 31.6%)	27.9% (24.8%, 31.1%)	30.9% (26.6%, 35.5%)		27.1% (24.0%, 30.5%)	31.3% (27.4%, 35.6%)
	6.5-6.9%	5.3% (4.2%, 6.7%)	6.0% (4.6%, 7.8%)	3.8% (2.4%, 6.0%)		2.7% (1.8%, 4.2%)	9.1% (7.0%, 11.8%)
	≥7.0%	3.1% (2.2%, 4.3%)	3.6% (2.6%, 5.1%)	2.0% (1.1%, 3.9%)		1.2% (0.6%, 2.4%)	5.8% (4.1%, 8.3%)
HbA1c (%)		5.8 (5.8, 5.9)	5.8 (5.8, 5.9)	5.8 (5.8, 5.9)		5.7 (5.7, 5.8)	5.9 (5.9, 6.0)
Hypertension		32.4% (29.8%, 35.1%)	35.3% (32.3%, 38.5%)	26.4% (22.4%, 30.8%)		16.1% (13.7%, 18.8%)	56.3% (51.9%, 60.6%)
BP category	Normal BP	54.8% (51.9%, 57.7%)	53.7% (50.4%, 57.0%)	57.0% (52.1%, 61.7%)		71.9% (68.5%, 75.0%)	29.7% (25.8%, 34.0%)
	High-normal BP	15.6% (13.6%, 17.7%)	14.6% (12.4%, 17.1%)	17.6% (14.3%, 21.4%)		14.1% (11.8%, 16.8%)	17.7% (14.7%, 21.1%)
	Grade 1 hypertension	16.3% (14.4%, 18.4%)	16.9% (14.6%, 19.5%)	15.1% (12.0%, 18.9%)		9.0% (7.3%, 11.2%)	27.0% (23.3%, 31.0%)
	Grade 2 hypertension	13.3% (11.6%, 15.3%)	14.8% (12.6%, 17.3%)	10.4% (7.9%, 13.5%)		5.0% (3.6%, 6.8%)	25.7% (22.3%, 29.4%)
Systolic BP (mmHg)		119.5 (119.0, 120.5)	120.0 (119.0, 121.5)	119.0 (118.0, 120.5)		120.0 (119.0, 121.0)	137.0 (135.0, 139.0)
Diastolic BP (mmHg)		76.5 (76.0, 77.5)	78.0 (77.5, 79.0)	74.0 (73.0, 75.0)		77.0 (76.5, 78.0)	86.5 (85.0, 88.0)

Footnotes: Values are presented as percentages (for categorical variables) or medians (for continuous variables). *CD4 counts are only reported among people living with HIV, excluding 5 people with missing CD4 count (N = 289). †BMI categories were created using BMI for age Z-scores among adolescents (<19 years) with WHO references and absolute BMI thresholds among adults (≥19 years). Mod/severe underweight = Z-score <-2 or BMI < 17kg/m2; mild underweight = Z-score ≥-2 & <-1 or BMI 17-18.49kg/m2; normal weight = Z-score ≥-1 & ≤+1 or BMI 18.5-24.9; overweight = Z-score >+1 & ≤+2 or BMI 25-29.9 kg/m2; obese = Z-score >+2 or BMI >30.0kg/m2. ‡Chronic lung disease was defined as either a previous diagnosis, or an obstructive or mixed defect on pre-bronchodilator spirometry (less than lower limit of normal using Global Lung Initiative 'Other' reference standard). 80.8% (n=1582) participants had an adequate quality spirometry, those missing spirometry traces were categorized as not having a chronic lung disease (unless self-reported). §Anaemia was categorised using age and sex-specific thresholds recommended by WHO (supplementary methods). , ll Stunting was only calculated among adolescents, using WHO reference standards. Mild stunting was defined as a height of age Z score of <-1 and ≥-2; moderate stunting as Z score of <-2 and ≥-3 and severe stunting as Z score <-3. **Abbreviations**: BMI = body mass index; BP = blood pressure; Hb = haemoglobin; HbA1c = glycosylated haemoglobin; HTN = hypertension; N = number.

Table J: Chronic conditions among TB household contacts, stratified by site (N = 1958)

Condition	Level	Overall	Mozambique	Tanzania	Zimbabwe
**All participants**		**N = 1958**	**N = 680**	**N = 650**	**N = 628**
HIV		15.0% (13.2%, 17.0%)	15.7% (13.0%, 19.0%)	12.9% (10.2%, 16.3%)	16.4% (13.0%, 20.4%)
CD4 category	≥500cells/uL	63.5% (57.7%, 68.9%)	69.5% (60.3%, 77.4%)	68.5% (57.7%, 77.7%)	52.9% (43.0%, 62.6%)
	200-499cells/uL	32.1% (27.0%, 37.7%)	26.7% (19.3%, 35.6%)	27.0% (18.5%, 37.5%)	42.2% (32.9%, 52.0%)
	<200cells/uL	4.4% (2.6%, 7.4%)	3.8% (1.4%, 9.8%)	4.5% (1.7%, 11.2%)	4.9% (2.1%, 10.9%)
CD4 (cells / uL)		584.0 (546.0, 616.0)	618.0 (565.0, 686.0)	633.0 (601.0, 708.0)	502.0 (449.0, 560.0)
BMI category	Moderate/severe underweight	3.9% (3.0%, 4.9%)	4.6% (3.1%, 6.7%)	3.8% (2.5%, 5.8%)	3.2% (2.1%, 4.9%)
	Mild underweight	13.5% (12.0%, 15.2%)	15.1% (12.6%, 18.1%)	16.5% (13.7%, 19.7%)	8.8% (6.5%, 11.6%)
	Healthy weight	54.5% (52.2%, 56.9%)	54.7% (50.6%, 58.8%)	52.5% (48.3%, 56.6%)	56.5% (52.4%, 60.5%)
	Overweight	17.4% (15.7%, 19.2%)	16.0% (13.3%, 19.2%)	16.2% (13.5%, 19.2%)	20.1% (17.0%, 23.6%)
	Obese	10.7% (9.4%, 12.2%)	9.6% (7.5%, 12.1%)	11.1% (8.8%, 13.9%)	11.5% (9.2%, 14.2%)
Chronic lung disease		10.3% (8.9%, 11.9%)	12.0% (9.6%, 14.8%)	11.3% (9.0%, 14.2%)	7.5% (5.3%, 10.5%)
Anaemia		17.7% (15.9%, 19.7%)	30.0% (26.3%, 34.0%)	11.2% (9.0%, 14.0%)	11.1% (8.5%, 14.5%)
Anaemia category	None	82.3% (80.3%, 84.1%)	70.0% (66.0%, 73.7%)	88.8% (86.0%, 91.0%)	88.9% (85.5%, 91.5%)
	Mild anaemia	11.5% (10.1%, 13.1%)	19.4% (16.5%, 22.7%)	7.2% (5.5%, 9.5%)	7.3% (5.4%, 9.9%)
	Moderate anaemia	5.6% (4.6%, 6.9%)	9.4% (7.2%, 12.2%)	3.7% (2.4%, 5.5%)	3.5% (2.2%, 5.5%)
	Severe anaemia	0.6% (0.3%, 1.2%)	1.2% (0.5%, 2.9%)	0.3% (0.1%, 1.2%)	0.3% (0.1%, 1.3%)
Hb (g/dL)		135.0 (134.0, 136.0)	127.0 (126.0, 129.0)	137.0 (136.0, 139.0)	138.0 (137.0, 140.0)
**Adolescents**		**N = 602**	**N = 221**	**N = 203**	**N = 178**
Stunting	Normal	56.6% (52.2%, 61.0%)	74.7% (68.8%, 79.8%)	30.5% (24.3%, 37.6%)	64.0% (56.4%, 71.1%)
	Mild stunting	26.7% (23.5%, 30.3%)	19.9% (15.4%, 25.4%)	32.0% (26.2%, 38.4%)	29.2% (23.1%, 36.2%)
	Moderate stunting	12.8% (10.2%, 15.9%)	5.4% (3.1%, 9.3%)	27.6% (21.9%, 34.1%)	5.1% (2.5%, 10.0%)
	Severe stunting	3.8% (2.5%, 5.8%)		9.9% (6.3%, 15.0%)	1.7% (0.5%, 5.2%)
**Adults**		**N = 1356**	**N = 459**	**N = 447**	**N = 450**
Diabetes		9.4% (7.9%, 11.1%)	7.8% (5.7%, 10.7%)	11.9% (8.8%, 15.7%)	8.4% (6.3%, 11.2%)
HbA1c category	<6.0%	62.8% (59.8%, 65.6%)	67.8% (62.4%, 72.7%)	61.1% (56.0%, 65.9%)	59.3% (54.1%, 64.4%)
	6.0-6.4%	28.8% (26.2%, 31.6%)	25.7% (21.4%, 30.5%)	27.3% (23.1%, 31.9%)	33.6% (28.9%, 38.5%)
	6.5-6.9%	5.3% (4.2%, 6.7%)	5.2% (3.6%, 7.6%)	6.0% (4.1%, 8.9%)	4.7% (3.1%, 7.0%)
	≥7.0%	3.1% (2.2%, 4.3%)	1.3% (0.6%, 2.9%)	5.6% (3.5%, 8.7%)	2.4% (1.4%, 4.3%)
HbA1c (%)		5.8 (5.8, 5.9)	5.8 (5.8, 5.9)	5.8 (5.8, 5.9)	5.8 (5.8, 5.9)
Hypertension		32.4% (29.8%, 35.1%)	30.7% (26.7%, 35.1%)	30.2% (25.8%, 35.0%)	36.2% (31.6%, 41.2%)
BP category	Normal BP	54.8% (51.9%, 57.7%)	60.6% (55.8%, 65.2%)	52.1% (47.0%, 57.2%)	51.6% (46.4%, 56.7%)
	High-normal BP	15.6% (13.6%, 17.7%)	12.0% (9.1%, 15.5%)	18.6% (15.1%, 22.6%)	16.2% (12.9%, 20.2%)
	Grade 1 HTN	16.3% (14.4%, 18.4%)	15.5% (12.4%, 19.2%)	15.0% (11.8%, 18.8%)	18.4% (15.1%, 22.4%)
	Grade 2 HTN	13.3% (11.6%, 15.3%)	12.0% (9.3%, 15.2%)	14.3% (11.3%, 18.0%)	13.8% (10.9%, 17.3%)
Systolic BP (mmHg)		119.5 (119.0, 120.5)	119.0 (117.5, 121.0)	119.5 (119.0, 121.5)	121.0 (120.0, 123.5)
Diastolic BP (mmHg)		76.5 (76.0, 77.5)	71.5 (70.5, 73.5)	78.0 (77.5, 79.0)	78.0 (78.0, 79.5)

Footnotes: Values are presented as percentages (for categorical variables) or medians (for continuous variables), with 95% confidence intervals. 'Prevalent' disease is defined as either known or screening detected. *CD4 counts are only reported among people living with HIV, excluding 5 people with missing CD4 count (N = 293). †BMI categories were created using BMI for age Z-scores among adolescents (<19 years) with WHO references and absolute BMI thresholds among adults (≥19 years). Mod/severe underweight = Z-score <-2 or BMI < 17kg/m2; mild underweight = Z-score ≥-2 & <-1 or BMI 17-18.49kg/m2; normal weight = Z-score ≥-1 & ≤+1 or BMI 18.5-24.9; overweight = Z-score >+1 & ≤+2 or BMI 25-29.9 kg/m2; obese = Z-score >+2 or BMI >30.0kg/m2. ‡Chronic lung disease was defined as either a previous diagnosis, or an obstructive or mixed defect on pre-bronchodilator spirometry (less than lower limit of normal using Global Lung Initiative 'Other' reference standard). 80.8% (n=1582) participants had an adequate quality spirometry, those missing spirometry traces were categorized as not having a chronic lung disease (unless self-reported). §Anaemia was categorised using age and sex-specific thresholds recommended by WHO (supplementary methods). ll Stunting was only calculated among adolescents, using WHO reference standards. Mild stunting was defined as a height of age Z-score of <-1 and ≥-2; moderate stunting as Z-score of <-2 and ≥-3 and severe stunting as Z-score <-3.
Abbreviations: BMI = body mass index; BP = blood pressure; Hb = haemoglobin; HbA1c = glycosylated haemoglobin; HTN = hypertension; N = number; WHO = World Health Organization.

Figure B: Prevalence of chronic conditions among TB household contacts stratified by age category and sex (N = 1958)

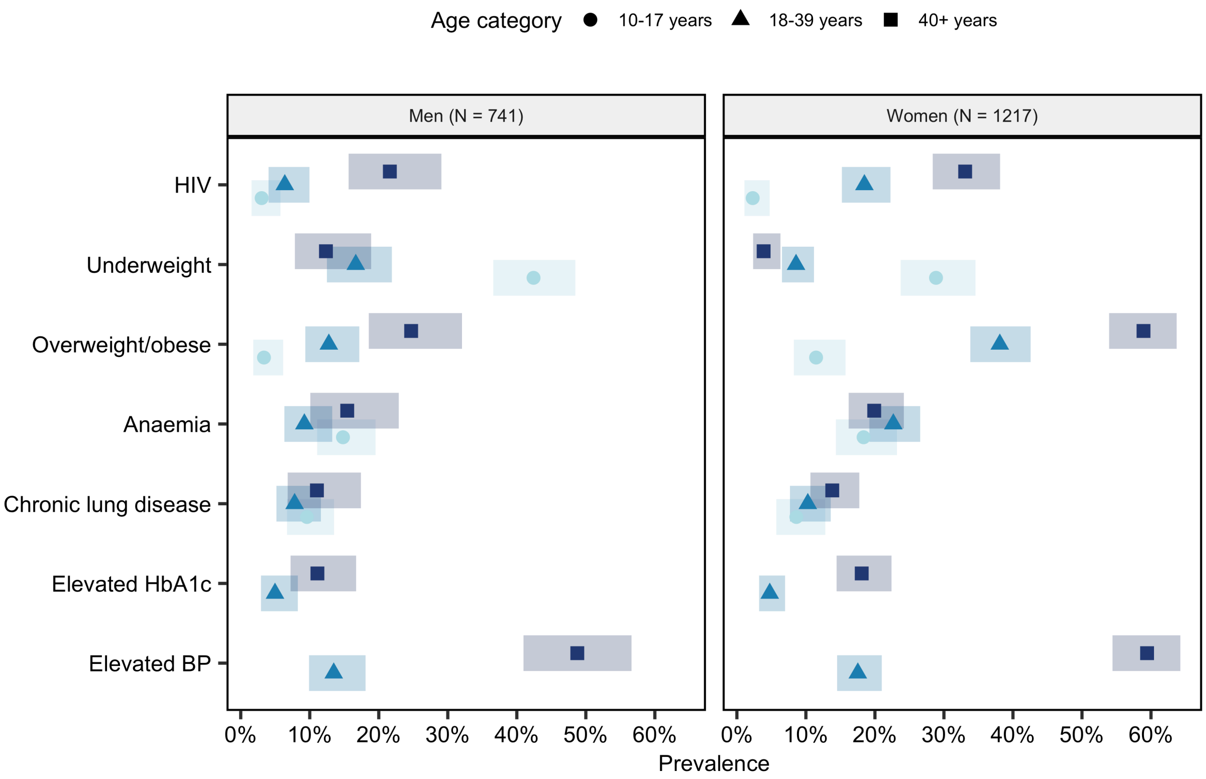

Figure C: Prevalence of chronic conditions among TB household contacts stratified by age category, sex and study site (N = 1958)

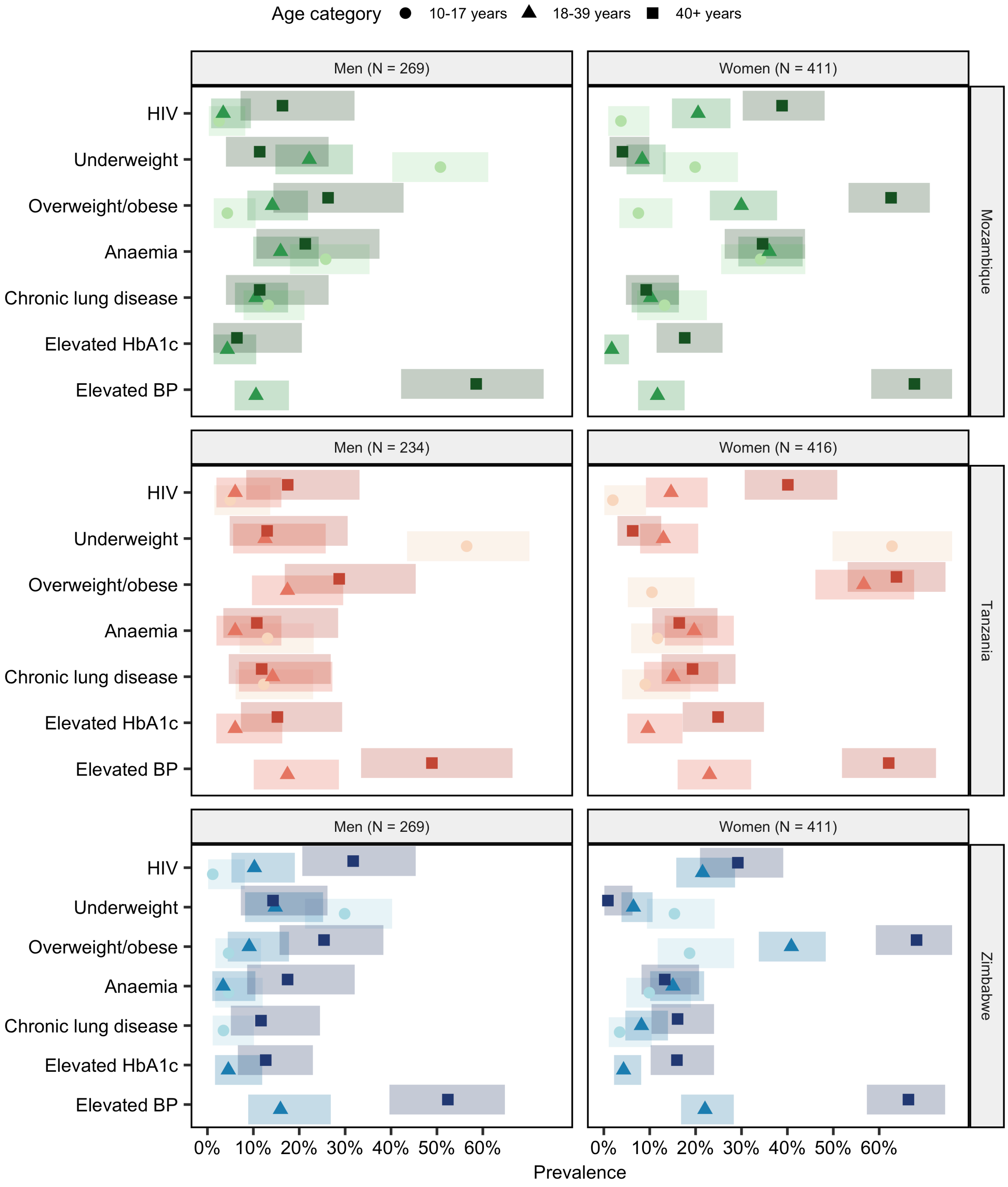

Table K: Site specific age-standardised prevalence of chronic conditions

	Mozambique		Tanzania		Zimbabwe	
**Condition**	**Women**	**Men**	**Women**	**Men**	**Women**	**Men**
HIV	23.5% (19.1%, 27.9%)	9.0% (4.6%, 13.3%)	16.0% (12.6%, 19.5%)	12.7% (3.4%, 21.9%)	19.0% (14.7%, 23.3%)	17.0% (11.1%, 22.9%)
Diabetes	8.8% (5.6%, 12.0%)	6.6% (2.2%, 11.0%)	14.0% (9.8%, 18.1%)	8.0% (3.8%, 12.1%)	9.7% (6.0%, 13.3%)	8.3% (4.1%, 12.5%)
Hypertension	39.4% (34.2%, 44.5%)	35.2% (27.7%, 42.7%)	33.7% (29.0%, 38.4%)	27.7% (20.3%, 35.1%)	44.4% (39.1%, 49.6%)	35.7% (27.6%, 43.8%)
Anaemia	33.0% (28.5%, 37.6%)	17.6% (12.3%, 22.8%)	12.5% (9.2%, 15.8%)	6.7% (3.7%, 9.6%)	12.7% (9.0%, 16.4%)	9.0% (4.9%, 13.1%)
Underweight	7.5% (5.3%, 9.6%)	31.5% (26.6%, 36.4%)	15.5% (12.6%, 18.4%)	20.8% (11.6%, 29.9%)	6.0% (3.9%, 8.1%)	17.6% (12.0%, 23.1%)
Overweight/Obese	35.5% (31.2%, 39.8%)	17.4% (11.7%, 23.0%)	38.3% (33.4%, 43.3%)	12.7% (8.2%, 17.1%)	47.4% (42.4%, 52.3%)	15.8% (11.5%, 20.1%)
Chronic lung disease	9.5% (6.6%, 12.4%)	10.9% (6.8%, 15.0%)	11.0% (7.8%, 14.2%)	16.0% (7.0%, 24.9%)	10.2% (6.8%, 13.6%)	5.0% (1.4%, 8.7%)

Footnotes: Age-standardized to World Health Organization reference population

Figure D: Association between HbA1c, systolic BP and diastolic BP, and age

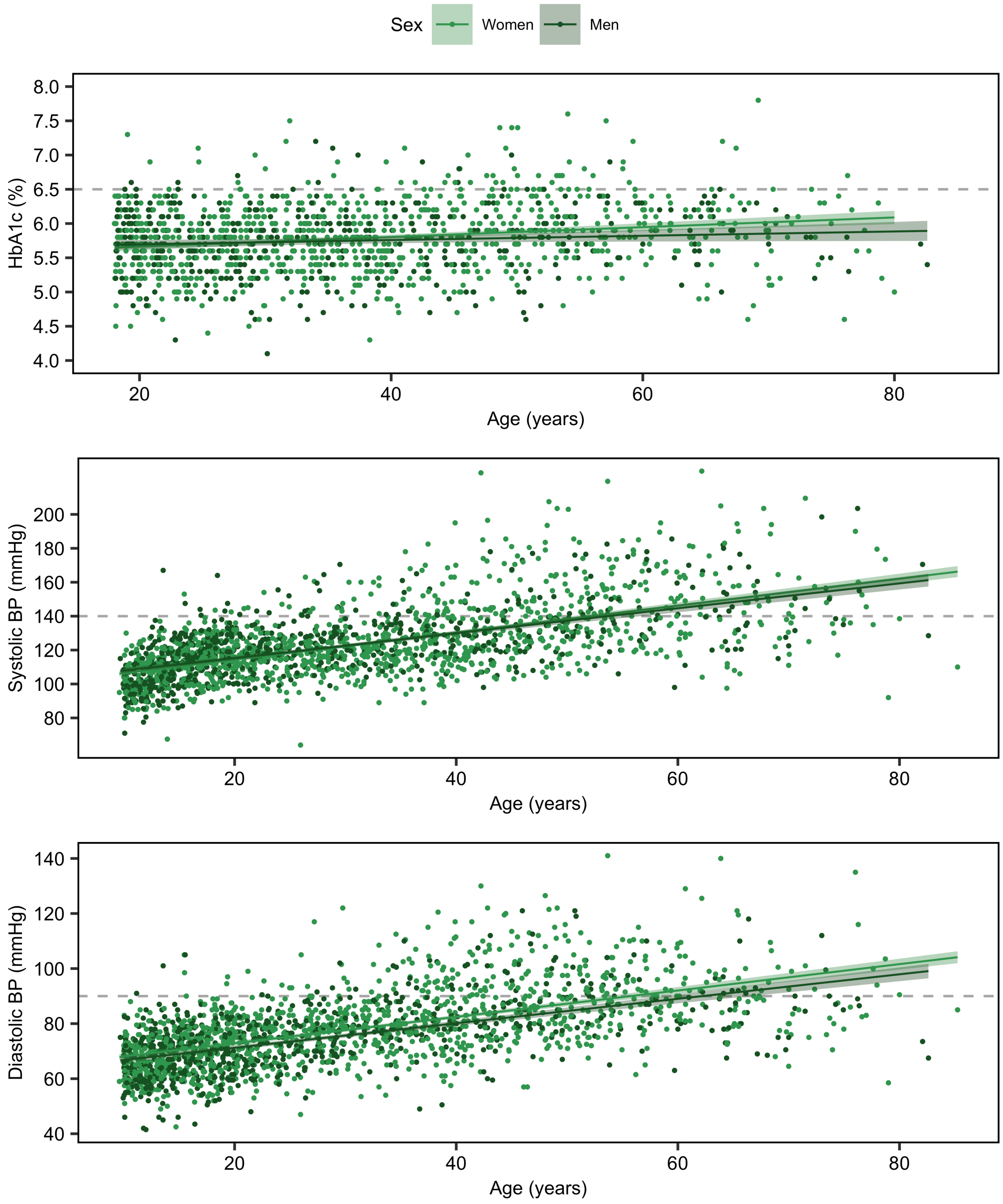

Footnotes: 21 HbA1c results >8.0% not displayed.

Figure E: Association between HbA1c, systolic and diastolic BP and BMI category

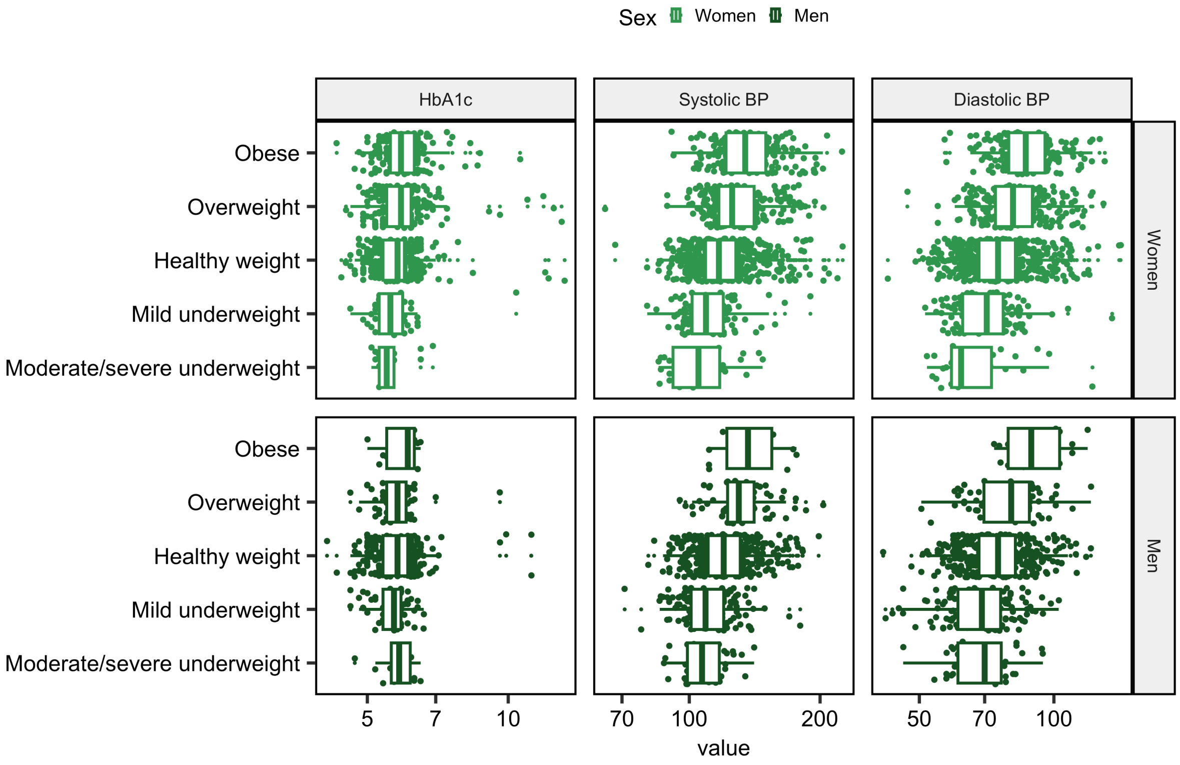

Figure F: BMI distribution by age

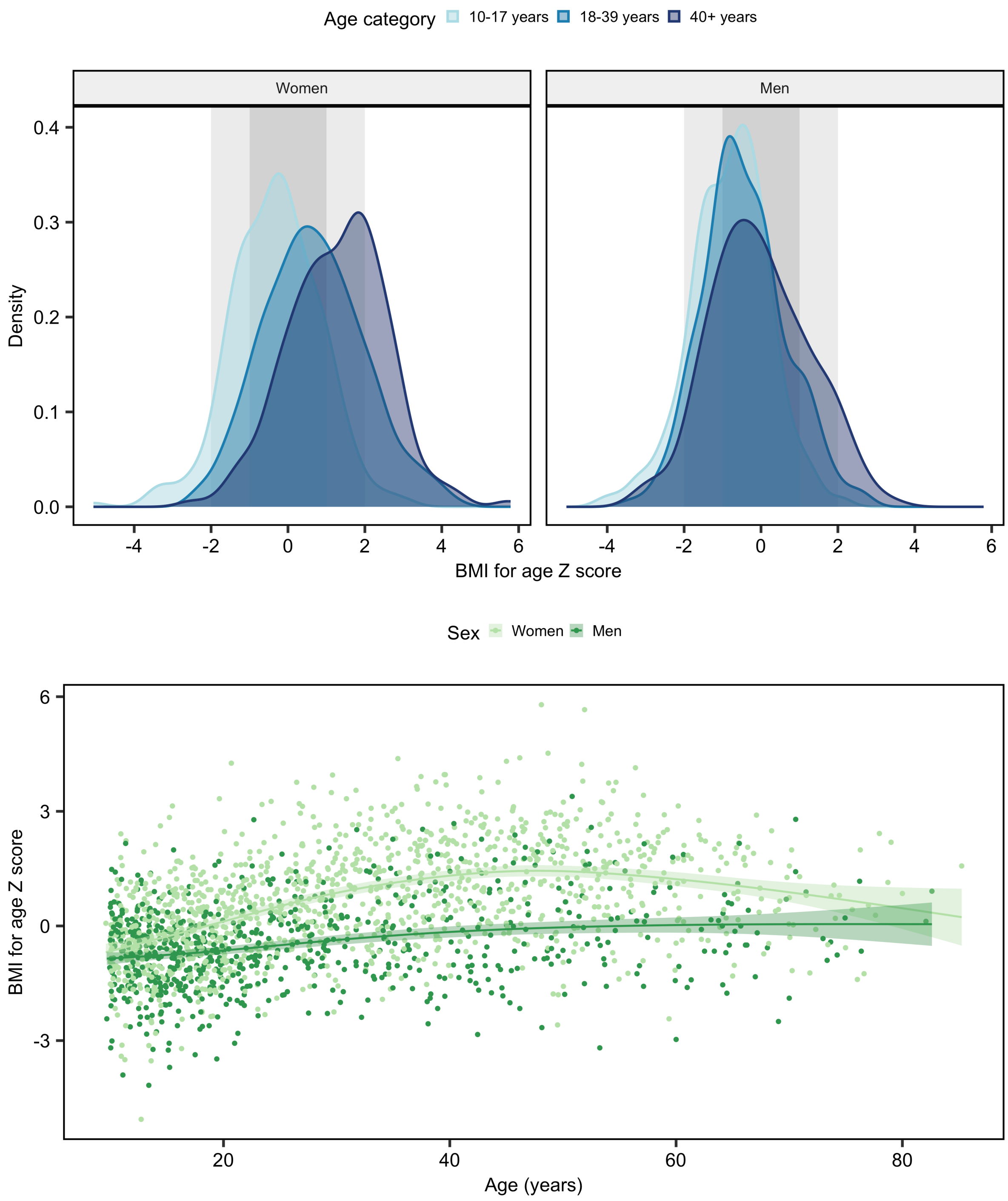

Table L: Prevalence of HIV among household contacts, stratified by HIV status of the person with TB (‘index case’)

		HIV negative index case		HIV positive index case	
Condition	Level	n/N	Estimate (95%CI)	n/N	Estimate (95%CI)
HIV	Overall prevalence	121/1235	9.8% (8.0%, 12.0%)	143/568	25.2% (21.2%, 29.6%)
	Known	110/1235	8.9% (7.2%, 11.0%)	127/568	22.4% (18.6%, 26.6%)
	Screening detected	11/1235	0.9% (0.5%, 1.7%)	16/568	2.8% (1.7%, 4.6%)

Table M: Cascade of care for HIV, diabetes and hypertension

Stratum	Condition	Total	Known		Treated		Controlled	
		N	N	Prevalence	N	Prevalence	N	Prevalence
Overall	HIV	278	255	91.7% (87.7%, 94.5%)	240	95.2% (91.8%, 97.3%)	205	85.4% (80.1%, 89.5%)
	Diabetes	127	26	20.5% (14.4%, 28.2%)	18	69.2% (49.1%, 84.0%)	10	55.6% (32.7%, 76.3%)
	Hypertension	439	145	33.0% (28.8%, 37.6%)	49	33.8% (26.7%, 41.6%)	9	18.4% (9.8%, 31.8%)
Women	HIV	225	207	92.0% (87.7%, 94.9%)	196	96.1% (92.3%, 98.0%)	172	87.8% (82.1%, 91.8%)
	Diabetes	95	17	17.9% (11.4%, 26.9%)	11	64.7% (39.9%, 83.5%)	5	45.5% (19.9%, 73.6%)
	Hypertension	322	127	39.4% (34.3%, 44.9%)	42	33.1% (25.6%, 41.5%)	8	19.0% (9.8%, 33.8%)
Men	HIV	53	48	90.6% (78.5%, 96.2%)	44	91.7% (79.4%, 96.9%)	33	75.0% (59.2%, 86.1%)
	Diabetes	32	9	28.1% (14.8%, 46.9%)	7	77.8% (39.9%, 94.9%)	5	71.4% (30.6%, 93.4%)
	Hypertension	117	18	15.4% (10.0%, 23.0%)	18	38.9% (19.5%, 62.5%)	1	14.3% (1.9%, 59.0%)
Zimbabwe	HIV	102	95	93.1% (86.4%, 96.7%)	88	93.6% (86.2%, 97.2%)	74	84.1% (73.6%, 90.9%)
	Diabetes	38	10	26.3% (14.5%, 42.9%)	5	50.0% (21.4%, 78.6%)	4	80.0% (28.6%, 97.6%)
	Hypertension	163	66	40.5% (33.6%, 47.8%)	29	43.9% (33.1%, 55.3%)	29	13.8% (5.2%, 32.0%)
Mozambique	HIV	98	93	94.9% (86.3%, 98.2%)	88	95.7% (88.9%, 98.4%)	75	85.2% (75.9%, 91.4%)
	Diabetes	36	10	27.8% (15.7%, 44.2%)	10	100.0% (100.0%, 100.0%)	6	60.0% (28.3%, 85.0%)
	Hypertension	141	57	40.4% (32.7%, 48.7%)	9	15.8% (8.3%, 28.0%)	2	22.2% (5.5%, 58.5%)
Tanzania	HIV	78	67	85.9% (76.1%, 92.1%)	64	97.0% (88.2%, 99.3%)	56	87.5% (76.8%, 93.7%)
	Diabetes	53	6	11.3% (5.2%, 23.0%)	3	50.0% (15.9%, 84.1%)	0	0.0% (0.0%, 0.0%)
	Hypertension	135	22	16.3% (10.7%, 24.0%)	11	50.0% (30.8%, 69.2%)	3	27.3% (8.9%, 59.1%)

Figure G: Cascade of care for HIV, diabetes and hypertension, stratified by study site

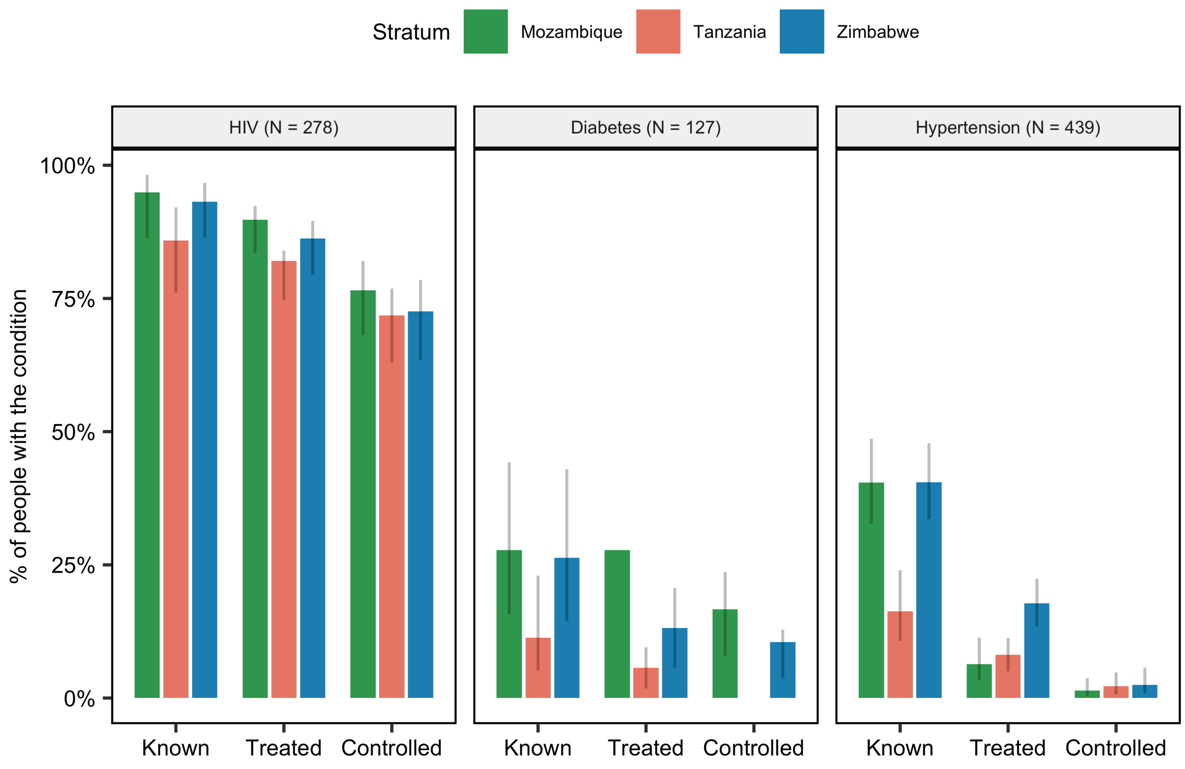

Table N: Prevalence of chronic conditions at household level (N=786 households)

**Characteristic**	**Overall**, N = 786	**Mozambique**, N = 269	**Tanzania**, N = 264	**Zimbabwe**, N = 253
HIV	245 (31%)	92 (34%)	77 (29%)	76 (30%)
Diabetes	119 (15%)	36 (13%)	45 (17%)	38 (15%)
Hypertension	343 (44%)	121 (45%)	105 (40%)	117 (46%)
Either diabetes or hypertension	379 (48%)	133 (49%)	119 (45%)	127 (50%)
Underweight	252 (32%)	98 (36%)	92 (35%)	62 (25%)
Stunting	87 (11%)	12 (4.5%)	63 (24%)	12 (4.7%)
Overweight/obesity	418 (53%)	134 (50%)	141 (53%)	143 (57%)

Footnotes: Presented as n (%).

Figure H: Overlap of underweight, stunting and overweight/obesity at household level (N = 786 households)

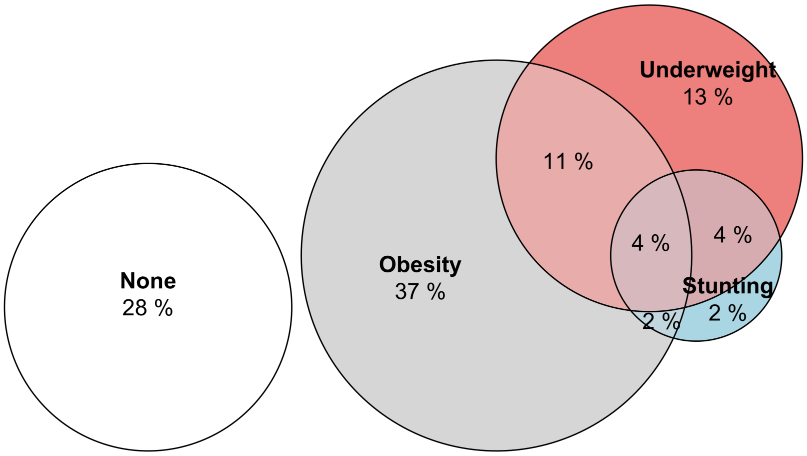

Additional tables added at peer review

Table O: Demographic characteristics of TB household contacts in Tanzania, stratified by residence area (N = 649*)

Characteristic	**Overall**, N = 649	**Rural**, N = 268[1]	**Peri-Urban**, N = 129	**Urban**, N = 252
**Sex**				
Women	416 (64%)	178 (66%)	81 (63%)	157 (62%)
Men	233 (36%)	90 (34%)	48 (37%)	95 (38%)
**Age, years**	29 (16, 44)	29 (15, 45)	31 (15, 44)	29 (17, 43)
**Age category**				
10-17 years	203 (31%)	84 (31%)	45 (35%)	74 (29%)
18-39 years	235 (36%)	97 (36%)	36 (28%)	102 (40%)
40+ years	211 (33%)	87 (32%)	48 (37%)	76 (30%)
**Highest educational level***				
None or primary school	471 (75%)	215 (81%)	100 (78%)	156 (67%)
At least secondary school	156 (25%)	50 (19%)	29 (22%)	77 (33%)
**Pregnant†**	14 (3.4%)	8 (4.5%)	1 (1.2%)	5 (3.2%)
**Smoking status**				
Non smoker	611 (94%)	258 (96%)	123 (95%)	230 (91%)
Smoker (current and/or former)	38 (5.9%)	10 (3.7%)	6 (4.7%)	22 (8.7%)
**Pack years smoking**	1.5 (0.5, 4.2)	3.1 (1.5, 4.5)	1.9 (1.1, 4.4)	0.6 (0.3, 2.5)
**Alcohol consumption**				
Never drunk alcohol	424 (65%)	179 (67%)	85 (66%)	160 (63%)
Alcohol, AUDIT-C negative	124 (19%)	43 (16%)	24 (19%)	57 (23%)
Alcohol, AUDIT-C positive	101 (16%)	46 (17%)	20 (16%)	35 (14%)
**Insufficient food‡**	38 (5.9%)	19 (7.1%)	5 (3.9%)	14 (5.6%)
**Known HIV**	70 (11%)	29 (11%)	11 (8.5%)	30 (12%)
**On ART**	67 (97%)	28 (100%)	11 (100%)	28 (93%)
**Known diabetes**	6 (0.9%)	1 (0.4%)	4 (3.1%)	1 (0.4%)
**Known hypertension**	22 (3.4%)	6 (2.2%)	6 (4.7%)	10 (4.0%)
**Previous TB**	25 (3.9%)	14 (5.2%)	6 (4.7%)	5 (2.0%)
**Relationship to index case**				
Spouse	123 (19%)	55 (21%)	18 (14%)	50 (20%)
Parent	165 (25%)	85 (32%)	32 (25%)	48 (19%)
Sibling	118 (18%)	39 (15%)	26 (20%)	53 (21%)
Child	91 (14%)	34 (13%)	20 (16%)	37 (15%)
Other	152 (23%)	55 (21%)	33 (26%)	64 (25%)

Footnotes: Presented a number (percentage) or median (interquartile range. 1-person missing residence area not shown in table. *Educational level not known for 22 participants. † The denominator for pregnancy is the number of women. ‡ Insufficient food was defined as participants as answering yes to 'was there any day in the past six months where you did not have enough food'. **Abbreviations**: AUDIT-C = alcohol use identification test, short form; TB = tuberculosis.

Table P: Chronic conditions among TB household contacts contacts in Tanzania, stratified by residence area (N = 649*)

Condition	Level	Rural	Peri-Urban	Urban
**All participants**		**N = 268**	**N = 129**	**N = 252**
HIV		12.7% (8.4%, 18.7%)	12.4% (7.3%, 20.3%)	13.5% (9.5%, 18.9%)
CD4 category*	≥500cells/uL	77.1% (59.8%, 88.5%)	66.7% (40.2%, 85.6%)	61.1% (43.5%, 76.3%)
	200-499cells/uL	17.1% (8.2%, 32.5%)	33.3% (14.4%, 59.8%)	33.3% (19.4%, 51.0%)
	<200cells/uL	5.7% (1.5%, 19.0%)		5.6% (1.4%, 20.1%)
CD4 (cells / uL)*		678.0 (601.0, 759.0)	652.0 (462.0, 863.0)	571.0 (430.0, 661.0)
BMI category	Moderate/severe underweight	3.7% (2.0%, 7.0%)	4.7% (2.1%, 9.9%)	3.6% (1.7%, 7.5%)
	Mild underweight	16.8% (12.1%, 22.8%)	17.8% (11.8%, 26.0%)	15.5% (11.8%, 20.0%)
	Healthy weight	56.3% (50.2%, 62.3%)	56.6% (47.3%, 65.5%)	46.4% (39.5%, 53.5%)
	Overweight	13.4% (9.6%, 18.5%)	14.0% (8.8%, 21.3%)	19.8% (15.6%, 24.9%)
	Obese	9.7% (6.8%, 13.7%)	7.0% (3.7%, 12.8%)	14.7% (10.3%, 20.5%)
Chronic lung disease		10.4% (7.2%, 15.0%)	10.6% (6.2%, 17.4%)	12.8% (8.8%, 18.2%)
Anaemia		13.4% (9.6%, 18.4%)	10.9% (6.4%, 17.9%)	9.1% (6.1%, 13.5%)
Anaemia category	None	86.6% (81.6%, 90.4%)	89.1% (82.1%, 93.6%)	90.9% (86.5%, 93.9%)
	Mild anaemia	9.7% (6.6%, 14.1%)	5.4% (2.6%, 10.8%)	5.6% (3.3%, 9.1%)
	Moderate anaemia	3.7% (1.8%, 7.4%)	4.7% (2.2%, 9.7%)	3.2% (1.6%, 6.2%)
	Severe anaemia		0.8% (0.1%, 5.3%)	0.4% (0.1%, 2.8%)
Hb (g/dL)		136.0 (134.0, 140.0)	136.0 (134.0, 139.0)	139.0 (136.0, 142.0)
**Adolescents**		**N = 84**	**N = 45**	**N = 74**
Stunting	Normal	26.2% (17.9%, 36.6%)	31.1% (18.7%, 47.1%)	35.1% (24.3%, 47.8%)
	Mild stunting	25.0% (18.2%, 33.3%)	42.2% (28.5%, 57.3%)	33.8% (24.0%, 45.2%)
	Moderate stunting	36.9% (28.3%, 46.5%)	24.4% (14.4%, 38.3%)	18.9% (11.0%, 30.6%)
	Severe stunting	11.9% (6.6%, 20.6%)	2.2% (0.3%, 15.2%)	12.2% (6.2%, 22.4%)
**Adults**		**N = 184**	**N = 84**	**N = 178**
Diabetes		10.9% (6.7%, 17.1%)	21.4% (12.7%, 33.9%)	8.4% (5.0%, 13.8%)
HbA1c category	<6.0%	61.4% (53.1%, 69.1%)	61.9% (49.0%, 73.3%)	60.7% (53.2%, 67.7%)
	6.0-6.4%	27.7% (21.0%, 35.6%)	17.9% (10.4%, 28.9%)	30.9% (24.4%, 38.3%)
	6.5-6.9%	6.0% (3.4%, 10.4%)	8.3% (3.1%, 20.4%)	5.1% (2.7%, 9.2%)
	≥7.0%	4.9% (2.1%, 11.0%)	11.9% (6.2%, 21.5%)	3.4% (1.3%, 8.4%)
HbA1c (%)		5.8 (5.8, 5.9)	5.8 (5.6, 6.1)	5.8 (5.7, 5.9)
Hypertension		33.7% (26.5%, 41.8%)	35.7% (26.6%, 46.0%)	24.2% (17.8%, 31.9%)
BP category	Normal BP	48.4% (40.0%, 56.9%)	46.4% (35.4%, 57.8%)	58.4% (50.4%, 66.0%)
	High-normal BP	19.0% (13.6%, 25.9%)	19.0% (12.2%, 28.4%)	18.0% (12.7%, 24.9%)
	Grade 1 hypertension	17.9% (12.9%, 24.5%)	16.7% (9.8%, 26.9%)	11.2% (7.2%, 17.0%)
	Grade 2 hypertension	14.7% (10.1%, 20.9%)	17.9% (11.2%, 27.3%)	12.4% (8.1%, 18.5%)
Systolic BP (mmHg)		119.5 (118.0, 122.5)	120.5 (116.5, 125.5)	119.0 (118.0, 122.5)
Diastolic BP (mmHg)		78.5 (77.0, 79.5)	77.5 (75.0, 80.5)	78.0 (77.0, 79.5)

Footnotes: Presented a prevalence estimate (for categorical variables) or medians (for continuous variables) with 95% confidence interval. 1-person missing residence area not shown in table. 'Prevalent' disease is defined as either known or screening detected. *CD4 counts are only reported among people living with HIV. †BMI categories were created using BMI for age Z-scores among adolescents (<19 years) with WHO references and absolute BMI thresholds among adults (≥19 years). Mod/severe underweight = Z-score <-2 or BMI < 17kg/m2; mild underweight = Z-score ≥-2 & <-1 or BMI 17-18.49kg/m2; normal weight = Z-score ≥-1 & ≤+1 or BMI 18.5-24.9; overweight = Z-score >+1 & ≤+2 or BMI 25-29.9 kg/m2; obese = Z-score >+2 or BMI >30.0kg/m2. ‡Chronic lung disease was defined as either a previous diagnosis, or an obstructive or mixed defect on pre-bronchodilator spirometry (less than lower limit of normal using Global Lung Initiative 'Other' reference standard). 83% participants had an adequate quality spirometry, those missing spirometry traces were categorized as not having a chronic lung disease (unless self-reported). §Anaemia was categorised using age and sex-specific thresholds recommended by WHO (supplementary methods). , ll Stunting was only calculated among adolescents, using WHO reference standards. Mild stunting was defined as a height of age Z-score of <-1 and ≥-2; moderate stunting as Z-score of <-2 and ≥-3 and severe stunting as Z-score <-3. **Abbreviations**: BMI = body mass index; BP = blood pressure; Hb = haemoglobin; HbA1c = glycosylated haemoglobin; HTN = hypertension; N = number; WHO = World Health Organization.

References

1 Smith S-J, Gray DM, MacGinty RP, *et al.* Choosing the Better Global Lung Initiative 2012 Equation in South African Population Groups. *Am J Respir Crit Care Med* 2020; **202**: 1724, 7.

2 Graham BL, Steenbruggen I, Barjaktarevic IZ, *et al.* Standardization of spirometry 2019 update an official American Thoracic Society and European Respiratory Society technical statement. *American Journal of Respiratory and Critical Care Medicine* 2019; **200**: E70, 88.

3 World Health Organization. Haemoglobin concentrations for the diagnosis of anaemia and assessment of severity. Geneva, Switzerland: World Health Organization (WHO), 2011.

4 World Health Organization. Guideline for the pharmacological treatment of hypertension in adults. Geneva: World Health Organization, 2021 https://iris.who.int/bitstream/handle/10665/344424/9789240033986-eng.pdf (accessed March 17, 2024).

5 World Health Organization. HEARTS Technical package for cardiovascular disease management in primary health care: risk based CVD management. Geneva: World Health Organization, 2018 https://iris.who.int/bitstream/handle/10665/333221/9789240001367-eng.pdf?sequence=1 (accessed March 17, 2024).

6 Bush K, Kivlahan DR, McDonell MB, Fihn SD, Bradley KA. The AUDIT alcohol consumption questions (AUDIT-C): an effective brief screening test for problem drinking. Ambulatory Care Quality Improvement Project (ACQUIP). Alcohol Use Disorders Identification Test. *Arch Intern Med* 1998; **158**: 1789, 95.

7 Household crowding. In: WHO Housing and Health Guidelines. World Health Organization, 2018. https://www.ncbi.nlm.nih.gov/books/NBK535289/ (accessed Dec 29, 2023).

8 PHIA Project. Mozambique population-based HIV impact assessment (INSIDA 2021). 2021 https://phia.icap.columbia.edu/mozambique-summary-sheet-en-pt-2021/ (accessed March 16, 2024).

9 Tanzania Summary Sheet 2016-2017. PHIA Project. https://phia.icap.columbia.edu/tanzania-summary-sheet/ (accessed March 16, 2024).

10 Ministry of Health and Child Care (MOHCC). Zimbabwe Population-based HIV impact assessment 2020 (ZIMPHIA 2020): Final Report. Harare, Zimbabwe: MOHCC, 2021.

11 World Health Organization. Global Tuberculosis Report 2020. Geneva: World Health Organization, 2020.

12 Sun H, Saeedi P, Karuranga S, *et al.* IDF Diabetes Atlas: Global, regional and country-level diabetes prevalence estimates for 2021 and projections for 2045. *Diabetes Research and Clinical Practice* 2022; **183**: 109119.

13 Global Health Observatory. Prevalence of hypertension among adults aged 30-79 years. https://www.who.int/data/gho/data/indicators/indicator-details/GHO/prevalence-of-hypertension-among-adults-aged-30-79-years (accessed March 16, 2024).

14 Global Nutrition Report | Country Nutrition Profiles - Global Nutrition Report. https://globalnutritionreport.org/resources/nutrition-profiles/africa/ (accessed Dec 20, 2023).

15 Badawi A, Gregg B, Vasileva D. Systematic analysis for the relationship between obesity and tuberculosis. *Public Health* 2020; **186**: 246, 56.

16 Gelaw Y, Getaneh Z, Melku M. Anemia as a risk factor for tuberculosis: a systematic review and meta-analysis. *Environ Health Prev Med* 2021; **26**: 13.
